# New Strategies Using Antibody Combinations to Increase Cancer Treatment Effectiveness

**DOI:** 10.3389/fimmu.2017.01804

**Published:** 2017-12-21

**Authors:** Isabel Corraliza-Gorjón, Beatriz Somovilla-Crespo, Silvia Santamaria, Jose A. Garcia-Sanz, Leonor Kremer

**Affiliations:** ^1^Department of Immunology and Oncology, Centro Nacional de Biotecnologia (CNB-CSIC), Madrid, Spain; ^2^Department of Cellular and Molecular Medicine, Centro de Investigaciones Biologicas (CIB-CSIC), Madrid, Spain

**Keywords:** cancer, antibody combinations, oncology, therapeutic antibodies, immunotherapy

## Abstract

Antibodies have proven their high value in antitumor therapy over the last two decades. They are currently being used as the first-choice to treat some of the most frequent metastatic cancers, like HER2^+^ breast cancers or colorectal cancers, currently treated with trastuzumab (Herceptin) and bevacizumab (Avastin), respectively. The impressive therapeutic success of antibodies inhibiting immune checkpoints has extended the use of therapeutic antibodies to previously unanticipated tumor types. These anti-immune checkpoint antibodies allowed the cure of patients devoid of other therapeutic options, through the recovery of the patient’s own immune response against the tumor. In this review, we describe how the antibody-based therapies will evolve, including the use of antibodies in combinations, their main characteristics, advantages, and how they could contribute to significantly increase the chances of success in cancer therapy. Indeed, novel combinations will consist of mixtures of antibodies against either different epitopes of the same molecule or different targets on the same tumor cell; bispecific or multispecific antibodies able of simultaneously binding tumor cells, immune cells or extracellular molecules; immunomodulatory antibodies; antibody-based molecules, including fusion proteins between a ligand or a receptor domain and the IgG Fab or Fc fragments; autologous or heterologous cells; and different formats of vaccines. Through complementary mechanisms of action, these combinations could contribute to elude the current limitations of a single antibody which recognizes only one particular epitope. These combinations may allow the simultaneous attack of the cancer cells by using the help of the own immune cells and exerting wider therapeutic effects, based on a more specific, fast, and robust response, trying to mimic the action of the immune system.

## Introduction

Nowadays, the therapeutic activity of antibodies in oncology has been widely demonstrated (1–6), being these proteins, after chemotherapy, radiotherapy, and small molecule inhibitors, one of the most used drugs for oncological treatments (7, 8). Most of the antibodies used on antitumor immuno-therapies had positive health effects as long as the antibody is present in the patient’s blood. The clinical use of antibodies directed against antigens not present on the tumor cells, but on cells of the immune system (i.e., anti-immune checkpoint antibodies), evidenced the beneficial effects of the treatment, which persisted even after it was finished (9, 10). These findings allowed to demonstrate that the anti-checkpoint antibodies were able to reprogram the organism’s response, re-directing the antitumor immune response, and skewing the balance on the tumor microenvironment toward immune destruction of the tumor. Thus, allowing to envisage a cure for cancer.

The aim of this review is to discuss the information on the possible anti-cancer treatments using monoclonal antibodies (mAbs; in clinical trials or already in the market) in combinations, either with other antibodies or with other biological agents (11–18). The clinical trials are mentioned throughout this review only as examples of the different types of combinations being currently analyzed for cancer treatment. Thus, for most of the studies details and results will be shown on Supplementary Tables S1 and S2. The use of radiation therapy, chemotherapy, small-molecule compounds, or stem cell autotransplants will be mentioned only, if the information is strictly necessary in our attempt to dissect the reasons behind the use of these combinations. Similarly, there is no specific section describing combinations including checkpoint inhibitory mAbs, since this subject has been recently reviewed (19–24), including the reviews by Xu-Monette and Young and Aris et al., in this issue (25, 26). Similarly, combinations directed against cancer stem cells will not be discussed since they have been treated elsewhere in this research topic (27).

Antibodies are generated by the immune system’s adaptive arm to defend the organism from pathogens and malignant cells. The antibodies are basically secreted molecules involved in mediating interactions on the extracellular compartment; they are made by a variable part that gives its binding specificity, and a constant region that is able to interact with other molecules or cells of the innate and adaptive immune system, to give them molecular information regarding their interaction with antigen. Thus, it should come to no surprise that antibodies’ functions promote health and that treatments based on antibodies might, therefore, be curative (6, 28, 29).

**Figure 1:**
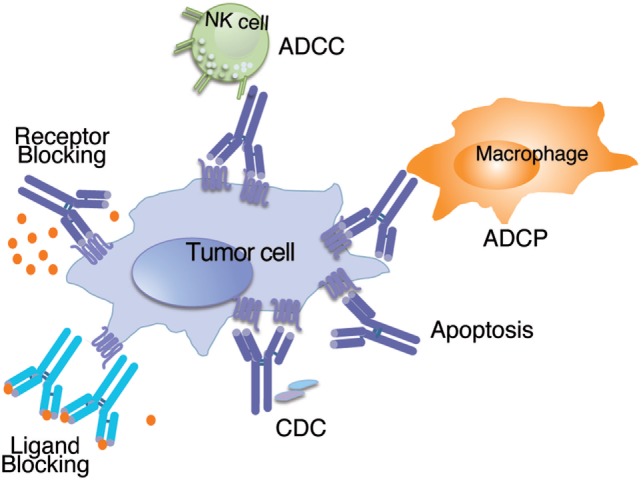
Schematic representation of the mechanisms of action used by naked antibodies to inhibit tumor growth. Naked antibodies can inhibit tumor growth through effector functions such as ADCC (antibody-dependent cell-cytotoxicity) where the antibody bound to the tumor antigen is recognized by the natural killer (NK) cell and triggers cytotoxic activity; can also trigger antibody-dependent cell-phagocytosis (ADCP) when the antibody bound to the tumor antigen opsonizes the cell and activates phagocytic cells; the antibody can also fix complement after binding to the tumor cell, and trigger complement-dependent cytotoxicity (CDC). Conversely, naked antibodies can kill the tumor cells by interfering with important signal pathways, either by binding to the ligand (Ligand Blocking) or by binding to the receptor (Receptor Blocking). In addition, they can trigger direct apoptosis after binding to an antigen on the tumor cell surface.

Very few therapeutic antibodies are able to directly kill the tumor cells, either by interacting with a signal pathway (i.e., as a receptor antagonist or sequestering the ligand), or by direct triggering of apoptosis. Most of them kill the tumor cells through the interaction with other molecules or cells of the immune system, acting as molecules mediating interactions on the extracellular compartment. Although they originated as receptors on the surface of cells from the acquired immune system, they became secreted on mature B cells, and through either engagement with Fc-receptor-bearing cells or by interaction with the complement system, they can exert a broad spectrum of effector functions, coordinating the immune response (see Figure 1).

A current goal of antitumor immune therapies is to trigger, from the beginning, all the possible host body defense mechanisms. Aiming to destroy, as early as possible, the highest number of tumor cells, decreasing the possibilities of the tumor developing escape mechanisms, to obtain a more effective therapy. The defense mechanisms include (i) to directly kill the tumor cells; (ii) to switch the immune system from an antitumor immunosuppressed status to another that allows to attack the tumor, i.e., through stimulating the secretion of cytokines or modulating cell to cell interactions; (iii) to attract the immune system cells to the tumor; (iv) to decrease the tumor-directed neo-vascularization; and (v) to inhibit migration, metalloprotease secretion, and tumor cell invasion, among others.

The current trends for the use of antibodies in oncology as therapeutic agents are to employ them either alone or, more often, as combinations with (i) cytotoxic agents; (ii) radiotherapy; (iii) molecularly targeted drugs interfering with tumor cell survival or proliferation; (iv) other antibodies against the same target; (v) other antibodies against molecules implicated in the same signaling pathway; (vi) other antibodies, each one of them specific for unrelated targets (including targets in immune system cells and neo-vascularization); (vii) vaccines or oncolytic virus; (viii) cells that would either act as immunogens or as effector cells; or (ix) adjuvants, liposomes, nanoparticles, etc.

For the treatment of cancer, the FDA and the EMA (United States and European Union Drug Administrations), have approved (or are reviewing) a total of 32 therapeutic antibodies or their derivatives. Interestingly, the number has doubled between 2012 and 2017 (Tables 1 and 2), concomitant with a 100% increase in phase III clinical trials using mAb on a similar time-period (30). Twenty of these antibodies are indicated for treatment of patients with solid tumors (Table 1), identifying 13 different targets; whereas 12 are indicated for neoplasias of hematological origin (Table 2), identifying eight different targets. The targets identified by these antibodies are described in Table 3. Other therapeutic antibodies, not yet approved, but mentioned on this review are summarized on Table 4.

**Table 1:** Antibodies approved (or in review) by the FDA and/or EMA for the clinical treatment of solid tumors.^a^

Approved indications	Target	International non-proprietary name	Brand name	Format	Proposed mechanism(s) of action	EU/US First approval year	Sponsor	Reference
Soft tissue sarcoma	PDGFRα	Olaratumab	Lartruvo	Human IgG1	Binds to PDGFR-α, blocks ligand binding and receptor signaling	2016/2016	Eli Lilly and Co.	(31–34)

Breast cancer	HER2	Pertuzumab	Perjeta	Humanized IgG1	Inhibits HER dimerization, prevents the formation of ligand-induced heterodimers of HER2 with other family members. Induces ADCC	2013/2012	Genentech	(35–38)

Breast cancer and gastric cancer	HER2	Trastuzumab	Herceptin	Humanized IgG1	Inhibits HER dimerization, prevents the formation of ligand-induced heterodimers of HER2 with other family members. Induces ADCC and phagocytosis	2000/1998	Genentech	(39–43)

Breast cancer	HER2	Ado-trastuzumab emtansine	Kadcyla	Humanized IgG1 conjugated to emtansine (ADC)	Inhibits HER dimerization, prevents the formation of ligand-induced heterodimers of HER2 with other family members. Induces ADCC and phagocytosis. Transports emtansine (microtubule inhibitor) to HER2-positive tumors	2013/2013	Genentech	(44–47)

HNSCC	Epidermal growth factor receptor (EGFR)	Necitumumab	Portrazza	Human IgG1	Binds to EGFR, blocks ligand binding and triggers EGFR internalization and degradation. Induces ADCC	2015/2015	Eli Lilly and Co.	(48–53)

Colorectal cancer	EGFR	Panitumumab	Vectibix	Human IgG2	Binds to EGFR, competitively inhibits the binding of its ligands, blocking receptor signaling	2007/2006	Amgen	(54–58)

HNSCC, Colorectal cancer	EGFR	Cetuximab	Erbitux	Chimeric IgG1	Binds to EGFR, blocks ligand binding and triggers EGFR internalization and degradation. Induces ADCC	2004/2004	ImClone LLC	(59–63)

Breast cancer, colorectal cancer, non-squamous NSCLC, RCC, cervix carcinoma, ovarian or fallopian tube cancer, primary peritoneal cancer, and glioblastoma	VEGFA	Bevacizumab	Avastin	Humanized IgG1	Binds to VEGFA, prevents interaction with its receptors and their subsequent activation	2005/2004	Genentech	(64–68)

NSCLC, gastric cancer, and colorectal cancer	VEGFR2	Ramucirumab	Cyramza	Human IgG1	Binds to VEGFR2, inhibits the binding of its ligands, blocking receptor signaling	2014/2014	Eli Lilly and Co.	(69–73)

NSCLC and urothelial carcinoma	PD-L1	Atezolizumab	Tecentriq	Humanized IgG1	Blocks the interaction of PD-L1 with programmed cell death protein 1 (PD-1) and CD80. Blocks the immune checkpoint inhibition. Contains a modified Fc region to limit ADCC or CDC	2017^b^/2016	Genentech	(74–78)

Urothelial carcinoma	PD-L1	Durvalumab	Imfinzi	Human IgG1	Blocks the interaction of PD-L1 with PD-1 and CD80. Blocks the immune checkpoint inhibition. Does not induce ADCC	NA/2017	AstraZeneca	(79–82)

Merkel cell carcinoma	PD-L1	Avelumab	Bavencio	Human IgG1	Blocks the interaction of PD-L1 with PD-1 and CD80. Blocks the immune checkpoint inhibition	2017^b^/2017	Merck/Pfizer	(83–86)

Melanoma, RCC, NSCLC, HNSCC, cHL and urothelial carcinoma	PD-1	Nivolumab	Opdivo	Human IgG4	Binds to PD-1, blocks its interaction with PD-L1 and PD-L2. Blocks the immune checkpoint inhibition. Does not induce ADCC	2015/2014	Ono Pharma (Japan)/Bristol-Myers Squibb (Worldwide)	(87–91)

Melanoma, NSCLC, HNSCC, and cHL	PD-1	Pembrolizumab	Keytruda	Humanized IgG4	Binds to PD-1, blocks its interaction with PD-L1 and PD-L2. Blocks the immune checkpoint inhibition. Does not induce ADCC	2015/2014	Merck	(92–96)

Melanoma	CTLA-4	Ipilimumab	Yervoy	Human IgG1	Binds to CTLA-4, blocks its interaction with CD80 and CD86, increasing T cell activation and proliferation	2011/2011	Bristol-Myers Squibb	(97–100)

Bone metastases from solid tumors, Increase of bone mass	RANK-L	Denosumab	Prolia Xgeva	Human IgG2	Binds to RANK-L, blocks its interaction with RANK, and prevents bone loss	2010/2010	Amgen	(101–105)

Colorectal cancer	IL-1α	MABp1^c^	Xilonix	Human IgG1	Binds to IL-1α, blocks its interaction with IL1-R	In review/NA	XBiotech	(106–108)

Neuroblastoma	GD2	Dinutuximab	Unituxin	Chimeric IgG1	Binds to the TAA GD2. Activates CDC and ADCC	2015^d, e^/2015	United Therapeutics	(109–112)

EpCAM^+^-Colon cancer	EpCAM	Edrecolomab	Panorex	Murine IgG2a	Engages immune effector cells. Activates CDC, ADCC, and phagocytosis	1995^d^^,^^e^/NA	GlaxoSmith Kline	(113–116)

EpCAM^+^-carcinomas related ascites	EpCAM/CD3	Catumaxomab	Removab	Rat/mouse bispecific monoclonal antibody	Attracts immune cells to the tumor proximity, promoting T cell activation and effector functions. Activates CDC, ADCC and phagocytosis	2009^e^/NA	Trion Pharma/Biotech	(117–121)

*^a^Adapted from Janice M. Reichert, PhD, The Antibody Society; last update July, 2017*.

*ADC, antibody–drug conjugate; ADCC, antibody-dependent cell-mediated cytotoxicity; CDC, complement-dependent cytotoxicity; cHL, classical Hodgkin lymphoma; HNSCC, head and neck squamous cell carcinoma; NSCLC, non-small cell lung cancer; RCC, renal cell carcinoma; TAA, tumor-associated antigen*.

*^b^Country-specific approval, 20 July 2017*.

*^c^International non-proprietary name pending*.

*^d^EMA initial authorization*.

*^e^Withdrawn or marketing discontinued for the first approved indication*.

*NA, not approved or in review in the EU, not approved or information on review status not available in the US*.

*Color shades corresponds to structurally related target molecules*.

**Table 2:** Antibodies approved (or in review) by the FDA and/or EMA for the clinical treatment of hematologic neoplasias.^a^

Approved indication	Target	International non-proprietary name	Brand name	Format	Proposed mechanism(s) of action	EU/US First approval year	Sponsor	Reference
ALL	CD19/CD3	Blinatumomab	Blincyto	Murine bispecific tandem scFv	Binds CD19 on tumor B cells and puts them in close contact with T cells through the CD3 (TCR complex), activates them, and results in redirected tumor cell lysis	2015/2014	Amgen	(122–126)

CLL and follicular lymphoma	CD20	Obinutuzumab	Gazyva Gazyvaro	Humanized IgG1; Glycoengineered	Lyses B cells by effector-cell recruitment. Enhanced CDC, ADCC, and ADCP, contains a modified Fc region with increased binding affinity for FcgammaRIII. Mutation (R7159) enhances apoptosis	2014/2013	Roche	(127–132)

CLL	CD20	Ofatumumab	Arzerra	Human IgG1	Binds to CD20 and engages immune effector cells, mediates B-cell lysis. Activates CDC, ADCC, and ADCP	2010/2009	Novartis	(133–136)

NHL	CD20	Tositumomab-^131^I	Bexxar	Murine IgG2a linked to ^131^I	Binds to CD20 and engages immune effector cells, mediates B-cell lysis. Activates CDC, ADCC, and ADCP, induces apoptosis. Ionizing radiation kills CD20^+^ cells	NA/2003^a^	GlaxoSmithKline	(137–140)

NHL	CD20	Ibritumomab-tiuxetan	Zevalin	Murine IgG1 linked to ^90^Y-tiuxetan	Binds to CD20, the tiuxetan moiety binds ^90^Y, the beta emission induces cell damage. Activates CDC, ADCC, and apoptosis	2004/2002	Spectrum Pharmaceuticals	(141–144)

NHL and CLL	CD20	Rituximab	MabThera-Rituxan	Chimeric IgG1	Binds to CD20 and engages immune effector cells, mediates B-cell lysis. Activates CDC, ADCC, and ADCP	1998/1997	Roche Biogen/Genentech	(145–150)

ALL	CD22	Inotuzumab ozogamicin	Besponsa	Humanized IgG4 linked to N-acetyl-gamma-calicheamicin (ADC)	Binds to CD22^++^ cells. After internalization, the toxin induces double-stranded DNA breaks and apoptosis	2017/2017	Pfizer	(151–155)

Hodgkin lymphoma and systemic anaplastic large cell lymphoma	CD30	Brentuximab vedotin	Adcetris	Chimeric IgG1 linked to monomethyl auristatin E (MMAE; ADC)	Binds to CD30^++^ cells. After internalization, the toxin MMAE, disrupts microtubules and induces apoptosis	2012/2011	Seattle Genetics	(156–161)

Acute myeloid leukemia	CD33	Gemtuzumab ozogamicin	Mylotarg	Humanized IgG4 linked to N-acetyl gamma calicheamicin (ADC)	Binds to CD33^+^ cells. After internalization, the toxin induces double-stranded DNA breaks and apoptosis. Does not activate ADCC	In review/in review; 2000^a^	Wyeth	(162–165)

Multiple myeloma	CD38	Daratumumab	Darzalex	Human IgG1	Binds to CD38^+^ cells. Activates CDC, ADCC, and ADCP	2016/2015	Janssen Biotech	(166–170)

Multiple myeloma	SLAMF7	Elotuzumab	Empliciti	Humanized IgG1	Binds to SLAMF7. Activates ADCC	2016/2015	Bristol-Myers Squibb	(171–176)

CLL	CD52	Alemtuzumab	Campath	Humanized IgG1	Binds to CD52^+^ lymphocytes. Activates ADCC and CDC	2001/2001	Genzyme	(177–179)

*^a^Adapted from Janice M. Reichert, PhD, The Antibody Society; last update July, 2017*.

*ADC, antibody–drug conjugate; ADCC, antibody-dependent cell-mediated cytotoxicity; ADCP, antibody-dependent cellular phagocytosis; ALL, acute lymphoblastic leukemia; CDC, complement-dependent cytotoxicity; CLL, chronic lymphoblastic leukemia; MAE, monomethyl auristatin E; NHL, non-Hodgkin lymphoma; scFv, single-chain variable fragment*.

*^b^Withdrawn or marketing discontinued for the first approved indication*.

*NA, not approved or in review in the EU, not approved or information on review status not available in the US*.

*Color shades corresponds to structurally related target molecules*.

**Table 3:** Characteristics of the main target molecules identified by therapeutic antibodies used in oncology.

Antibody target		Nature of the target	Function	Expression	Monoclonal antibody effects in cancer therapy	Reference

PDGFRα	Platelet-derived growth factor receptor alpha	Protein of the tyrosine kinase family	Cell proliferation, differentiation and migration	Ubiquitous, highly expressed on endothelial cells	Cell proliferation inhibition	(180, 181)

HER2	Human epidermal growth factor receptor 2	Glycoprotein of the tyrosine kinase family	Enhances cell proliferation and favors survival	Epithelial cells, highly expressed on many tumors	Cell proliferation inhibition	(182, 183)

EGFR	Epidermal growth factor receptor	Glycoprotein of the tyrosine kinase family	Cell proliferation and differentiation	Epithelial cells	Cell proliferation inhibition	(184, 185)

VEGFA	Vascular endothelial growth factor A	Glycoprotein of the PDGF/VEGF family	Proliferation and migration of endothelial cells	Hypoxic cells, highly expressed on many tumors	Angiogenesis inhibition	(186, 187)

VEGFR2	Vascular endothelial growth factor receptor 2	Cell surface receptor of the tyrosine kinase family	Proliferation and migration of endothelial cells	Vascular and lymphatic endothelial cells	Angiogenesis inhibition	(186, 187)

PD-L1	Programmed cell death-1 ligand 1	Protein of the immunoglobulin superfamily	Inhibits T cell activation and cytokine production	Myeloid and lymphoid lineage cells, highly expressed on certain cancer cells	Immune checkpoint inhibition	(188, 189)

PD-1	Programmed Cell Death-1	Protein of the immunoglobulin superfamily	Inhibits T cell activation and cytokine production	B and T lymphocytes	Immune checkpoint inhibition	(188, 189)

CTLA-4	Cytotoxic T-lymphocyte antigen 4	Protein of the immunoglobulin superfamily	Inhibits T cell activation and cytokine production	B and T lymphocytes	Immune checkpoint inhibition	(188–190)

RANK-L	Receptor activator of nuclear factor κB ligand	Ligand of the tumor necrosis factor superfamily	Activates osteoclast through NF-kappa B activation	Osteoblasts and T lymphocytes	Inhibition of bone destruction	(191–193)

IL-1α	Interleukin-1 alpha	Cytokine of the interleukin-1 family	Pleiotropic effects, including inflammatory response and apoptosis	Secreted by activated macrophages and monocytes	Cell growth inhibition and anti-inflammatory	(194)

GD2	Glycolipid disialoganglioside	Cell surface glycolipid receptor	Attachment of tumor cells to extracellular matrix	Nervous system cells and melanocytes, highly expressed on neuroblastomas and melanomas	Activates CDC and ADCC	(195, 196)

EpCAM	Epithelial cell adhesion molecule	Cell surface glycoprotein	Cell adhesion	Epithelial tissues, highly expressed on carcinomas	Activates CDC, ADCC, and ADCP	(188, 189, 197, 198)

CD3	CD3 subunit of the T cell receptor complex	Cell surface glycoprotein of the immunoglobulin superfamily	T cell receptor signal transduction	T lymphocytes	Activates CDC and ADCC	(188, 189)

CD19	B cell Receptor CD19	Surface antigen of the immunoglobulin superfamily	B cell differentiation and activation	B lymphocytes and DC	Activates CDC and ADCC	(188, 189)

CD20	B cell receptor CD20	Cell surface antigen of the MS4A family	B cell development and activation	B lymphocytes and a subset of T cells	Activates CDC, ADCC, and ADCP	(188, 189)

CD22	B cell receptor CD22	Surface antigen of the immunoglobulin superfamily	B cell signaling and adhesion	B lymphocytes	ADC	(188, 189)

CD30	Tumor necrosis factor receptor superfamily member 8	Cell surface antigen of the TNF-receptor superfamily	Pleiotropic effects, including lymph proliferation, differentiation, and activation	T and B lymphocytes and natural killer (NK) cells	ADC	(188, 189)

CD33	Platelet endothelial cell adhesion molecule	Cell surface lectin	Cell adhesion and apoptosis	Myeloid lineage cells	ADC	(188, 189)

CD38	ADP-ribosyl cyclase 1	Cell surface glycoprotein	Cell activation and adhesion	NK cells, DC, and macrophages, highly expressed on B cells and activated T cells	Activates CDC, ADCC, and ADCP	(188, 189)

SLAMF7	Signaling lymphocytic activation molecule family member 7	Cell surface receptor of the CS2 family	NK cells activation	T and B lymphocytes, NK cells, DC, and monocytes	Activates CDC and ADCC	(188, 189)

CD52	CAMPATH-1	Cell surface glycoprotein	Modulates lymphocytes activation and adhesion	T and B lymphocytes, NK cells, monocytes, macrophages, epithelial, and sperm cells	Activates CDC and ADCC	(188, 189)

*ADC, antibody–drug conjugate; ADCC, antibody-dependent cell-mediated cytotoxicity; ADCP, antibody-dependent cellular phagocytosis; CDC, complement-dependent cytotoxicity; DC, dendritic Cells; MS4A, membrane-spanning 4-domains subfamily A; NK cells, natural killer cells*.

*Color shades corresponds to structurally related target molecules*.

**Table 4:** Summary of the therapeutic antibodies not yet approved for clinical treatments.^a^

Antibody name	Molecular format	Company or Institute	Target/main characteristics	Reference/Clinical Trial Identifier
3H1 (CEA-Vac)	Mouse IgG1	Titan Pharmaceuticals	Anti-idiotype antibody that mimics an epitope of the carcinoembryonic antigen (CEA)	(199)/NCT00033748

5B1 (MVT-5873)	Human IgG1	MabVax Therapeutics	Carbohydrate determinant 19-9 (CA19-9, carbohydrate antigen sialyl-Lewis A)	(200)/NCT03118349

11D10 (TriAb)	Mouse IgG1	Titan Pharmaceuticals	Anti-idiotype antibody that mimics a human milk fat globule membrane epitope	(201)/NCT00033748, NCT00045617

A27.15	Mouse IgG1	University of Arizona	Anti-transferrin receptor (TfR) antibody that blocks the binding of transferrin	(202)/NCT00003082

Abagovomab	Mouse IgG1	CellControl Biomedical Laboratories; Menarini	Anti-idiotype antibody that mimics an epitope of the ovarian cancer tumor-associated antigen CA-125	(203)/NCT00058435, NCT01959672

Andecaliximab (GS-5745)	Humanized IgG4	Gilead Sciences	Anti-matrix metalloproteinase 9 antibody that inhibits its enzymatic activity	(204, 205)/NCT02864381

B-701	Human IgG1	BioClin Therapeutics	Fibroblast growth factor receptor 3. Antagonist	(206)/NCT03123055

Basiliximab (Simulect)	Chimeric mouse–human IgG1	Novartis; Cerimon Pharmaceuticals	Interleukin-2 receptor alpha-subunit (IL-2Ralpha, IL2Ra, CD25). Antagonist	(207)/NCT00626483

BMS-986148	ADC	Bristol-Myers Squibb	Mesothelin (MSLN). Antibody conjugated to an undisclosed cytotoxic drug	(208)/NCT02341625

BMS-986179	Bristol-Myers Squibb	Bristol-Myers Squibb	Ecto-5’-nucleotidase (CD73)	(209–211)/NCT02754141

BTH1704	Humanized IgG1	Cancer Research UK; Biothera	Mucin-1 (MUC1). Antagonist	(212)/NCT02132403

Cabiralizumab (FPA008)	Humanized IgG4	Five Prime Therapeutics; Bristol-Myers Squibb; Ono Pharmaceutical	Colony-stimulating factor 1 receptor (CSF1R). Antagonist	(213)/NCT02526017, NCT03158272

Canakinumab (ACZ885)	Fully human IgG1	Novartis Pharmaceuticals	Interleukin-1 beta (IL-1beta, IL-1b). Antagonist	(214)/NCT02900664

Carotuximab (TRC105)	Chimeric mouse-human IgG1	Roswell Park Cancer Institute; Santen Pharmaceutical; TRACON Pharmaceuticals	Endoglin (CD105). Inhibitor	(215)/NCT03181308

CC-90002	Humanized IgG	Inhibrx; Celgene Corporation	Leukocyte surface antigen CD47. Antagonist	(216)/NCT02367196

CDX-1401	Human antibody fusion protein	Ludwig Institute for Cancer Research; Celldex Therapeutics Inc	Dendritic and epithelial cell receptor DEC205. Antibody linked to the tumor-associated antigen NY-ESO-1	(217)/NCT02129075, NCT02495636

Cergutuzumab amunaleukin (CEA-IL2v, RG7813)	Immunocytokine	Roche	CEA. Antibody fused to a single IL-2 variant moiety with abolished CD25 binding	(218)/NCT02350673

CJM112	Fully human IgG1	Novartis	Interleukin-17A (IL-17, IL-17A). Antagonist	(219)/NCT03111992, NCT02900664

Conatumumab (AMG 655)	Fully human IgG1	Amgen; Takeda	Tumor necrosis factor-related apoptosis-inducing ligand receptor 2 (TRAIL-R2, DR5). Agonist	(220)/NCT01327612

Darleukin (L19-IL2)	Immunocytokine, fusion protein	Philogen; Bayer HealthCare Pharmaceuticals	Extra-domain B domain of fibronectin. A human single-chain variable fragment (scFv) antibody fragment fused to interleukin-2 (IL-2)	(221)/NCT02076633

Demcizumab (OMP-21M18)	Humanized IgG2	OncoMed Pharmaceuticals	Delta-like ligand 4 (DLL4). antagonist	(222)/NCT02722954

Drozitumab (PRO95780)	Fully human, IgG1	Genentech	TRAIL-R2 (DR5). Agonist	(223)/NCT00851136

E2.3	Mouse IgG1	Salk Institute	Anti-TfR antibody that blocks the binding of transferrin to the receptor	(202, 224)/NCT00003082

Emactuzumab (RO5509554)	Humanized IgG1	Roche	CSF1R (CD115). Antagonist	(225)/NCT02760797

EMD 525797 (DI17E6)	Humanized IgG2	EMD Serono; Merck Serono	Integrin alpha-V subunit (CD51). Antagonist	(226)/NCT01008475

Emibetuzumab (LY2875358)	Humanized IgG4	Eli Lilly	c-Met receptor tyrosine kinase (c-MET; MET; hepatocyte growth factor receptor; c-Met proto-oncogene)	(227)/NCT02082210

Epratuzumab (AMG 412)	Humanized IgG1	Immunomedics	Anti-CD22 antibody that mediates antibody-dependent cellular cytotoxicity (ADCC)	(228)/NCT00941928

Ficlatuzumab (AV-299, SCH 900105)	Human IgG1	AVEO Pharmaceuticals	Hepatocyte growth factor (HGF). Inhibitor	(229)/NCT02277197

Ganitumab (AMG 479)	Fully human IgG1	Amgen; NantWorks; Takeda	Insulin-like growth factor 1 receptor. Antagonist	(230)/NCT00788957, NCT01327612

GD2Bi-aATC (Hu3F8Bi-armed ATC)	Humanized bispecific	National Cancer Institute; Barbara Ann Karmanos Cancer Institute	CD3 and disialoganglioside GD2	(231, 232)/NCT02173093

Imalumab (BAX69)	Fully human	Cytokine PharmaSciences; Shire	Macrophage migration inhibitory factor. Inhibitor	(233)/NCT02448810

IMC-CS4 (LY3022855)	Human IgG1	ImClone Systems	CSF1R (C-FMS; CD115)	(234)/NCT03153410

Intetumumab (CNTO 95)	Fully human IgG1	Centocor; BeiGene	Anti-Integrin alpha-V subunit (CD51) antibody that blocks both alpha-v beta-3 and alpha-v beta-5 integrins	(235)/NCT00888043

Lirilumab	Humanized monoclonal antibody (mAb) IgG4	Bristol-Myers Squibb	KIR (killer-cell immunoglobulin-like receptors)	(236)/NCT01714739

m170	Mouse IgG1	University of California, Davis	MUC1	(237)/NCT00009750

MEDI3617	Human IgG1	MedImmune	Angiopoietin 2. Antagonist	(238)/NCT01248949, NCT02141542

Milatuzumab (hLL1)	Humanized IgG1	Immunomedics	CD74	(239)/NCT00989586

Mirvetuximab soravtansine (IMGN853)	Chimeric mouse-human, ADC	ImmunoGen	Folate receptor 1. Antibody conjugated to the maytansinoid DM4 (N2′-Deacetyl-N2′-(4-mercapto-4-methyl-1-oxopentyl)-maytansine)	(240)/NCT02606305

MM-111	Human bispecific	Merrimack Pharmaceuticals	ErbB receptors ErbB2 and ErbB3. Inhibitor	(241)/NCT01097460

MNRP1685A	Fully human IgG1	Genentech; Roche	Neuropilin-1. Inhibitor	(242)/NCT00954642

MOXR0916	Humanized IgG1	Genentech	OX40. Antagonist	(243)/NCT02410512

Navicixizumab (OMP-305B83)	Bispecific Humanized IgG2	OncoMed Pharmaceuticals	Delta-like ligand 4 (DLL4) and vascular endothelial growth factor A (VEGF). Inhibitor	(244)/NCT03030287, NCT02298387

Nimotuzumab (TheraCim hR3, BIOMAb EGFR, Theraloc)	Humanized IgG1	Center of Molecular Immunology; CIMYM	Epidermal growth factor receptor (EGFR). Inhibitor	(245)/NCT02947386

Otlertuzumab (TRU-016)	Recombinant single-chain polypeptide	Aptevo Therapeutics	CD37 Potential immunostimulatory and antineoplastic activities	(246)/NCT01317901

Parsatuzumab (MEGF0444A)	Humanized IgG1	Genentech	Epidermal growth factor-like domain 7. Inhibitor	(247)/NCT01399684

PD-0360324	Humanized IgG2	Pfizer	Cytokine CSF1 (CSF-1, macrophage colony-stimulating factor, M-CSF). Inhibitor	(248)/NCT02554812

PDR001	Humanized IgG4	Novartis	Programmed cell death protein 1 (PD-1). Inhibitor	(13)/NCT02900664, NCT03111992

PF-04518600	Fully human IgG2	Pfizer	OX40. Agonist	(249)/NCT02554812

Pidilizumab (CT-011, MDV9300)	Humanized IgG1	CureTech; Medivation	PD-1, Inhibitor	(250); NCT01067287

Relatlimab BMS-986016	Human mAb IgG4	Bristol-Myers Squibb	LAG-3 (human lymphocyte activation gene 3 protein) Potential immune checkpoint inhibitory and antineoplastic activities	(251)/NCT01968109, NCT02061761

Rilotumumab (AMG102)	Fully human IgG2	Amgen	Human hepatocyte growth factor (HGF, c-Met). Inhibitor	(252)/NCT00788957

RO6958688	Bispecific	Roche	CD3 and CEA. Inhibitor	(253, 254)/NCT02650713

RO7009789 (CP-870,893)	Fully human IgG2	Roche	CD40. Agonist	(255)/NCT02665416, NCT02760797

Rovalpituzumab tesirine (SC16LD6.5)	Humanized ADC	Stemcentrx	Delta-like protein 3 (DLL3). Antibody conjugated to tesirine, a pyrrolobenzodiazepine dimer	(256)/NCT03026166

SGN-LIV1A	Humanized ADC	Seattle Genetics	Zinc transporter LIV-1 (SLC39A6). Antibody conjugated to maleimidocaproylvaline-citrulline-p-aminobenzyloxycarbonyl-MMAE (vcMMAE)	(257)/NCT01969643

SS1 (dsFv) PE38 (CAT-5001)	Immunotoxin	National Institutes of Health (USA)	MSLN. Single-chain antibody linked to Pseudomonas exotoxin PE-38	(258)/NCT01051934

Tigatuzumab (CS-1008)	Humanized IgG1	Daiichi Sankyo Company; University of Alabama at Birmingham	TRAIL-R2 (DR5). Agonist	(259, 260)/NCT01307891

Urelumab (BMS-663513)	Fully human IgG4 mAb	Bristol-Myers Squibb	Anti-CD137 Potential immunostimulatory and antineoplastic activities	(261)/NCT01471210, NCT01775631, NCT02110082, NCT02253992

Utomilumab (PF-05082566)	Human IgG2	Pfizer	CD137 (4-1BB). Agonist	(262)/NCT02554812

Vanucizumab (RG7221)	Bispecific Humanized	Roche	Angiopoietin 2 (ANG2, ANGPT2) and vascular endothelial growth factor (VEGF). Inhibitor	(263)/NCT01688206, NCT02665416, NCT02715531

Varlilumab (CDX-1127)	Fully human IgG1	Celldex Therapeutics	CD27. Agonistic.	(16)/NCT02410512

Veltuzumab (IMMU-106, hA20)	Humanized	Immunomedics	Anti-CD20 antibody that triggers complement-dependent cell lysis and antibody-dependent cell-mediated cytotoxicity (ADCC)	(264)/NCT00989586

VGX-100	Fully human IgG1	Circadian Technologies Limited	Vascular endothelial growth factor C (VEGF-C or Flt4 ligand). Inhibitor	(265)/NCT01514123

*^a^Only clinical trials included in this review*.

From the antibodies approved (or under review) for the treatment of solid tumors, seven of them recognize tumor cell surface tyrosine kinase receptors involved in proliferation and survival pathways. These receptors (see Table 3) are PDGFRα (targeted by the antibody olaratumab), HER2 (pertuzumab, trastuzumab and emtansine, ado-trastuzumab) and epidermal growth factor receptor (EGFR; necitumumab, panitumumab, and cetuximab). Two antibodies inhibit tumor angiogenesis by binding to the soluble ligand VEGF (bevacizumab) or to the endothelial cell receptor VEGFR2 (ramucirumab). Six of the mAb disrupt inhibitory immune checkpoint signals by binding to the programmed cell death protein 1 (PD-1) receptor on the T cells (nivolumab and pembrolizumab), to PD-L1 on the tumor cells (atezolizumab, durvalumab, and avelumab) or to CTLA-4 on T cells (ipilimumab). Two of these mAb block the binding of cytokines that are involved in the growth of some tumors, including antibodies against RANK-L (denosumab) and IL-1α (MABp1). The last three mAb recognize antigens overexpressed on the surface of tumor cells. They identify GD2 (dinutuximab) and EpCAM (edrecolomab and catumaxomab) (Table 1).

From the antibodies approved (or under review) for the treatment of hematopoietic neoplasias, eight antibodies recognize B cell antigens. Among those, one recognizes CD19 (blinatumomab), five mAb recognize CD20 (obinutuzumab, ofatumumab, rituximab, ibritumomab tiuxetan, and 131I tositumomab), one mAb binds to CD22 (inotuzumab ozogamicin), and the last one recognizes CD52 (alemtuzumab). Other two antibodies identify antigens expressed by B cells and by other cells of the immune system, including an anti-CD30 mAb (shared between B and T cells, brentuximab vedotin) and an anti-SLAMF7 (present on activated B cells and natural killer (NK) cells among others, elotuzumab). In addition, there are two antibodies against other immune cells, an anti-CD33 (myeloid lineage, gemtuzumab ozogamicin); and the non-lineage-restricted CD38 (daratumumab) (Table 2).

We will describe in the following paragraphs a set of clinical trials using antibodies in combination for oncological treatments, giving a systematic description of the antibody combinations with biological agents and their rationale. Describing the current aims of antibody-mediated cancer therapy and to envisage where its future lies. In addition, there will be a section where the therapeutic effects and toxicities for selected clinical trials will be discussed, which will help us to envisage the future of therapeutic antibodies for cancer treatments. Before starting with this systematic analysis, we will describe, with one example, in this case for the treatment of GD2^+^-neuroblastomas, the complexity of the clinical trials being carried out.

## Evolution of Treatment Complexity with Antibody in Combinations

In this section, we will discuss, as an example, the use of anti GD2 antibodies for the treatment of GD2-positive solid tumors, including neuroblastoma (266–269). Near 50 clinical assays have been started using two different mouse antibodies and their corresponding chimeras or humanized antibodies. The clinical use of dinutuximab (ch14.18) was approved in 2015, whereas the therapeutic efficacy of the other antibody, 3F8 has been demonstrated with many patients (270–272). These antibodies, recognizing the neuroblastoma tumor-associated antigen GD2, are being used to (i) kill the tumor with either the naked antibody alone, apparently through Fc-mediated effector actions [antibody-dependent cell cytotoxicity (ADCC), complement-dependent cytotoxicity (CDC), and antibody-dependent cell phagocytosis (ADCP)] or apoptosis (NCT00002458, NCT00072358, NCT01418495, NCT01419834, NCT01704872, NCT02258815, NCT02743429); (ii) kill the tumor by the naked antibody in combination with chemotherapeutic agents (busulfan, carboplatin, cisplatin, cyclophosphamide, doxorubicin, etoposide, lomustine, melphalan, or vincristine), small molecule drugs (crizotinib), external radiation, and/or conventional surgery (NCT03098030, NCT03126916); (iii) directly transport a radioelement toward the tumor, by conjugating the radioelement to the anti-GD2 mAb. This will induce radiolysis of the tumor cells, minimizing the effects on normal cells (NCT00058370, NCT00445965, NCT03126916); (iv) use them in combination with agents that modify cell expression patterns, inhibiting proliferation and inducing cell differentiation and apoptosis [i.e., isotretinoin (13-cis retinoic acid or RA)] (NCT00003022, NCT00030719, NCT01183416, NCT01183429, NCT01183884, NCT01526603, NCT01711554, NCT02100930, NCT03033303); or (v) use them in combination with agents able to burst the host immune response against the tumor. These include, granulocyte-macrophage colony-stimulating factor (GM-CSF) or granulocyte colony-stimulating factor (G-CSF) (NCT01704716, NCT01767194, NCT02484443, NCT02502786, NCT03189706); which increase the number of innate immune response cells by triggering the proliferation of granulocytes and macrophages; increasing both innate and adaptive responses by inducing the maturation/proliferation of NK cells and T lymphocyte proliferation with interleukin-2 (IL-2) alone or in combination with GM-CSF and/or RA (NCT00005576, NCT00026312, NCT01041638, NCT01592045, NCT01662804, NCT02169609, NCT02641782); regulating the threshold of the immune response with an adjuvant, changing the secreted cytokine expression pattern of cells bearing certain pattern recognition receptors (i.e., beta glucan that binds the C-type lectin receptor Dectin-1) (NCT00037011, NCT00492167, NCT00089258); increasing the pool of cytotoxic cells able to fight the tumor with allogeneic NK cells (NCT00877110, NCT01857934, NCT02573896, NCT02650648); using *in vitro* activated T cells coated with bispecific OKT3-hu3F8 mAb, together with IL-2 and GM-CSF to redirect T lymphocyte cell lysis (NCT02173093); and combining the anti-GD2 antibody with nivolumab, an anti-immune checkpoint (PD-1) mAb able to block the immunosuppressor activity induced by the tumor (NCT02914405).

From these “basic” aims further combinations arose, for example one where the aim is to induce radiolysis of the tumor cells with ^131^I-3F8, simultaneously bursting the innate immune response with filgastrim (G-CSF), inhibiting neo-vascularization with bevacizumab (anti-VEGF), together with autologous stem cell rescue of irradiated patients (NCT00450827).

We believe that this example gave a rough idea of the complexity that clinical trials for one antibody (two in this case) can reach. The chimeric, human-murine, anti-GD2 mAb dinutuximab has been approved in combination with GM-CSF, IL-2, and retinoic acid for the treatment of pediatric patients with high-risk neuroblastoma (273). Interestingly, the overall survival and event-free survival of patients treated with dinutuximab increased 2 years when compared to standard treatment during phase III clinical trials (273).

## Combination of Antibodies with Non-Biological Agents

Chemotherapeutic drugs are cytotoxic agents affecting unspecifically cell proliferation and survival, which inhibit topoisomerases I or II (doxorubicin, etoposide, irinotecan, topotecan, etc.), produce DNA breaks interfering with DNA replication, RNA transcription and cell division through changes in DNA alkylation, DNA methylation, and DNA cross-linking or intercalating between base pairs in the DNA helix (busulfan, melphalan, cyclophosphamide, carboplatin, cisplatin, lomustine, thiotepa, etc.). These chemotherapeutic drugs are being used in combination with mAbs for many cancer treatments (274).

In addition to surgery, treatment with antibodies and external irradiation has also been used. Localized external irradiation allows, by destroying tumor cells, better exposure of the tumor antigens to the immune system cells, this combination is also working well and is being used in numerous clinical trials (275–279).

Small molecule drugs that inhibit molecular interactions or enzymatic activity of proteins involved in cell signaling, or inhibitors of protein kinases overexpressed in tumor cells (including erlotinib, ibrutinib, imatinib, lapatinib, olaparib, regorafenib, ruxolitinib, sorafenib, sunitinib, etc.), are also being used in combination with antibodies (280, 281). There are numerous examples of treatments with this type of combinations that, by simultaneously inhibiting ligand–receptor interactions and kinases belonging to the same signaling pathway, have led to very positive therapeutic results (282–286).

## Combination of Antibodies with Biological Agents

These are therapies that use a combination of antibodies or antibody-based molecules with other biological substances, for example, recombinant proteins, genetic material, virus, bacteria, and cells (16). Most of these strategies are designed to stimulate the host immune system to act against the cancer cells.

In the following paragraphs, we describe antibodies in combinations, where (i) one of the antibodies identifies a tumor-associated antigen (an antigen overexpressed in tumor cells), used either naked, as an antibody–drug conjugate (ADC) or as an immunotoxin; (ii) antibodies against the tumor cell are used in combination with cytokines or immunocytokines to burst the immune response against the tumor, or conversely use anti-cytokine antibodies when the expressed cytokines can be harmful for the antitumor response, aiming to disrupt their balance; (iii) the antibodies directly target the angiogenesis process, aiming to inhibit new vascularization required for tumor growth; (iv) the mAb can also be combined with effector cells to increase the immune response against the tumor; or (v) combined with antibodies against immunomodulatory or immunostimulatory proteins to disrupt the inhibitory signals sent by the tumor to the host immune system to inhibit the antitumor response. Although several of the examples we will describe could be included more than one subheading, each one of them is described only in one of them.

### Antibodies Against Tumor-Associated Antigens

The rationale of using antibodies as therapeutic agents was to kill the tumor cells either directly or through activating the patient’s immune system effector functions (ADCC, CDC, or phagocytosis) with antibodies specific for tumor-associated antigens. Mucin 1 (MUC-1), an antigen present on the surface of many adenocarcinomas, which is recognized by mAb m170 (237). This mAb has been used radiolabeled as ^111^In-m170 or ^90^Y-m170, in combination with chemotherapy and the immunosuppressor cyclosporine to treat patients with metastatic prostate cancer that did not respond to hormone therapy. This treatment was followed by peripheral stem cell transplantation (NCT00009750). The rationale is to kill the tumor with the combination of chemotherapy and the mAb coupled to the radioisotope in the presence of cyclosporine. Afterward peripheral stem cell transplantation will allow to refurbish the hematopoietic compartment.

Other approaches have been used on hematological neoplasias, one of them, a combination of two anti-transferrin receptor (TfR) antibodies A27.15 and E2.3 (202, 287) was used for the treatment of chronic myeloproliferative disorders (NCT00003082). The anti-TfR mAb block the binding of (Fe^3+^)2-transferrin to TfR, resulting in decreased tumor cell growth. Other targets used in hematopoietic malignancies are CD20 and CD74. In this case, a combination of the anti-CD20 mAb veltuzumab (IMMU-106) (288) and milatuzumab (anti-CD74) (239) was used to treat relapsed or refractory B cell non-Hodgkin lymphoma (NCT00989586). CD74, a surface receptor of the pro-inflammatory cytokine macrophage migration inhibitory factor (MIF) (289–291), is an MHC class II chaperone and an accessory-signaling molecule (292). Milatuzumab induces apoptosis, related to inhibition of CD74 activation by MIF, ADCC, or CDC (293), while veltuzumab triggers CDC and ADCC in cells that overexpress CD20 (288). Other combinations include rituximab (anti-CD20) in combination with CC-90002 (anti-CD47) (294, 295) for the treatment of advanced solid and hematologic cancers (NCT02367196). CC-90002 selectively binds to CD47 expressed on tumor cells, blocks CD47 interaction with signal regulatory protein alpha (SIRPa), a protein expressed on phagocytic cells, which prevents CD47/SIRPa-mediated signaling and abrogates the CD47/SIRPa-mediated inhibition of phagocytosis. The result is an induction of pro-phagocytic signaling, resulting in macrophage activation and the specific phagocytosis of tumor cells. In addition, CD47 signaling blockade activates both, an antitumor T lymphocyte immune response and T cell-mediated killing of CD47-expressing tumor cells. CD47, also called integrin-associated protein (IAP), is a tumor-associated antigen (TAA) expressed on normal, healthy hematopoietic stem cells (HSC) and overexpressed on the surface of a variety of cancer cells. Expression of CD47 and its interaction with SIRPa leads to the inhibition of macrophage activation and protects cancer cells from phagocytosis, resulting in cancer cell proliferation (294, 296–298).

### ADC and Immunotoxins

Some antitumor treatments use, rather than naked antibodies, antibody–toxin fusion proteins (immunotoxins) or antibodies linked to drugs (299–302). In some cases, to increase the cell-killing potential of antibodies, they can be covalently linked to potent cytotoxic or cytostatic agents, including small molecule drugs or inactive forms of a biological toxin. The antibody directs the toxin toward the tumor cell. When the cell endocytoses the ADC, it undergoes enzymatic cleavage and the drug is released, gets activated, and exerts its cytotoxic action, killing the tumor cell. The endocytic process works for antigens that can be internalized. Most of the current antibodies that are being used as ADC identify cell surface receptors that are efficiently endocytosed. However, many cell surface proteins are not internalized and a large amount of work is being carried out to develop alternatives, such as making an ADC where the antibody is coupled to the drug through a linker that can be cleaved by tumor cell-surface proteases, when in close contact with the tumor cell; conversely the antibody may carry a tumor receptor antagonist to direct it toward the tumor cell surface (303, 304).

One example includes a combination of an antitumor-associated antigen mAb and an ADC. Trastuzumab in combination with an antibody against the zinc transporter LIV-1 (SLC39A6) conjugated to the cytotoxic agent monomethyl auristatin E (MMAE) (257) for the treatment of patients with metastatic breast cancer (NCT01969643). In this particular combination, trastuzumab inhibits the tyr-kinase receptor HER2, while through the potent microtubule disrupting agent MMAE, which is coupled to the anti-LIV-1 antibody, induces cell cycle arrest in the G2/M phase and apoptosis of LIV-1^+^ cells (257). This type of combination can be made more potent by adding to the equation, antibodies against immune-checkpoints to burst antitumor immune responses. For example, the combination of nivolumab, ipilimumab, and rovalpituzumab tesirine has been used in extensive-stage small cell lung cancer (SCLC) (NCT03026166). Rovalpituzumab tesirine is an anti-delta-like protein 3 (DLL3) antibody conjugated to the cytotoxic pyrrolobenzodiazepine dimer D6.5 (256, 305). This antibody recognizes the membrane protein DLL3, which is overexpressed in certain tumors, binds to Notch receptors, and regulates Notch-mediated signaling (256). Thus, this combination should kill the cells overexpressing DLL3, while the anti-checkpoint antibodies nivolumab and ipilimumab redirect the host immune response to attack the tumor.

Another example shows the combination of mAb CR011, against the transmembrane protein GPNMB (glycoprotein non-metastatic B) coupled to MMAE (306). This ADC was used in combination with anti-PD-1 mAb nivolumab or pembrolizumab and varlilumab (307, 308), an agonistic anti-CD27 mAb, for the treatment of advanced melanoma (NCT02302339). The rationale is, in addition to targeting the GPNMB^+^ cells with the antibody-coupled to the toxin, the anti-PD-1 antibodies suppress the tumor-promoted inhibition of the antitumor immune response, while the anti-CD27 triggers an activation of the cytotoxic T lymphocytes (CTL).

The following example combines the ADC mirvetuximab soravtansine with either bevacizumab (VEGF) or pembrolizumab (PD-1) in primary peritoneal, fallopian tube, or endometrial cancer (NCT02606305). Mirvetuximab soravtansine is an immunoconjugate consisting of a folate receptor 1 (FOLR1) mAb (M9346A) conjugated to the cytotoxic maytansinoid DM4 (240). DM4 is released after internalization, binds to tubulin, and disrupts microtubule dynamics. FOLR1 is a member of the folate receptor family, overexpressed on a variety of epithelial-derived cancer cells (240, 309). Other studies combine nivolumab (anti-PD-1) with the ADC BMS-986148 (208, 310), composed of a mAb against the cell surface glycoprotein mesothelin (MSLN), conjugated to an as of yet undisclosed cytotoxic drug, for the treatment of mesothelioma, NSCLC, ovarian cancer, pancreatic cancer, and gastric cancer (NCT02341625). The rationale here is to block with the anti-PD-1 mAb the binding of PD-L1 (present on the tumor cells) to its receptor PD-1 (present on T cells), avoiding the suppression of antitumor responses triggered by the PD-L1/PD-1 interaction, while targeting mesothelin^+^ cells with the BMS-986148 mAb. Since the mAb is an ADC, upon internalization the cytotoxic agent kills the tumor cells. The mAb also activates ADCC. Another combination, for the treatment of MSLN-expressing NSCLC uses a combination of the anti-VEGF antibody bevacizumab with the single-chain anti-MSLN mAb SS1 (dsFv) linked to the exotoxin PE-38 from Pseudomonas (258) (NCT01051934). Since MSLN is not shed in significant amounts into the bloodstream, the dsFv-toxin can be concentrated onto the tumor cell surface. Once the dsFv toxin is internalized, the toxin is released and inactivates eukaryotic translation elongation factor 2, disrupting tumor cell protein synthesis. Concomitantly, the anti-VEGF antibody inhibits angiogenesis (258).

### Antibodies Combined with Cytokines and Immunocytokines

Another way to burst the host immune response against the tumor involves the use of either exogenous cytokines or fusion proteins that include a cytokine, administered either systemically or directly in the tumor. In some cases, due to cytokine toxicity, it could be envisaged to directly couple the cytokine to a mAb specific for a tumor-associated antigen, as a recombinant fusion protein (311, 312). These combinations allow, decreasing the dose, to reach higher local concentrations at the tumor site and to exert its therapeutic function avoiding systemic toxicity, while increasing the cytokine’s half-life, since it is coupled to the antibody, which prevents renal clearance (313).

In another example for the treatment of advanced or metastatic solid tumors, a combination of nivolumab and the Aldesleukin Prodrug NKTR-214 was used (NCT02983045). NKTR-214 is a recombinant human IL-2 conjugated to six releasable polyethylene glycol chains (PEG) (314). When the cytokine is released, binds to CD122 (IL-2 receptor beta subunit) and the mAb may act synergistically with NKTR-214 by blocking PD-1 activation through the mAb and simultaneously stimulating growth and cytotoxic activity against the tumor of the patient’s T and NK cells by the exogenous IL-2. The advantages of using this conjugated form of the IL-2 are that, on the one side, is released in a controlled way in the tumor’s proximity, avoiding systemic toxic effects; and on the other side, PEG conjugation prevents IL-2 binding to the IL2Ralpha subunit (and the subsequent activation of CD4-positive regulatory immunosuppressive T cells), while IL2Rbeta activation plays a key role on the proliferation and activation of effector T cells (314). In another clinical trial, the NKTR-214 immunocytokine was also administered, using a similar therapeutic strategy for the treatment of patients with metastatic urothelial bladder cancer or metastatic NSCLC, in combination with atezolizumab (74–76) (NCT03138889). Another trial for advanced or metastatic solid tumors expressing the carcinoembryonic antigen (CEA), combined atezolizumab and cergutuzumab amunaleukin [CEA-IL-2 variant (IL2v)] (218), alone or together with a pretreatment with the anti-CD20 mAb obinutuzumab (NCT02350673). The immunocytokine CEA-IL2v is a fusion protein between a recombinant IL2v, unable to bind CD25, fused to the C-terminus of a high affinity, bivalent CEA-specific antibody (218). The strategy is to inhibit the PD-1/PD-1L checkpoint, increasing locally IL-2 activity on the tumor cells, allowing its binding to CD122. A similar strategy uses nivolumab in combination with the superagonist ALT-803 for the treatment of advanced and unresectable NSCLC (NCT02523469). ALT-803 is a fusion protein containing a mutated IL-15 (IL-15N72D) cytokine and a soluble, dimeric IL-15 receptor alpha Fc fusion protein (IL-15Ra-Fc) (315). The rationale of this study is to suppress the signaling through negative checkpoints, while activating and increasing NK levels and memory CD8^+^ T cells, through the binding of ALT-803 to the IL-2/IL-15 receptor beta gamma chain, strengthening the patient’s immune response.

Another example is the anti-HER2 mAb trastuzumab, which has been used in combination with IL-12 in treating patients with recurrent solid tumors [breast cancer, endometrial carcinoma, gastric cancer, non-small cell lung cancer (NSCLC), SCLC, and ovarian epithelial cancer] (NCT00028535). IL-12 stimulates IFN-gamma production and enhances T- and NK-cell proliferation, differentiation, and activation (316). A more complex example combines two mAb–cytokine fusion proteins, consisting of L19, a human single-chain variable fragment directed against the extra-domain B (ED-B) of fibronectin, linked to either the human pro-inflammatory cytokine tumor necrosis factor alpha (TNFa, L19-TNF) or to human IL-2 (L19-IL-2). These combinations have been used on patients with malignant melanoma (317–320) (NCT02076633). The rationale of the trial is that the L19 moiety binds to the ED-B domain of a fibronectin isoform selectively expressed in the tumor neovasculature during neo-angiogenesis. TNFa may locally induce an immune response against ED-B^+^ tumor cells, while the IL-2 moiety may locally activate CTL, NK cells, and macrophages.

In another clinical trial, atezolizumab was combined with (i) ipilimumab, (ii) interferon alfa-2b, (iii) PEG-interferon Alfa-2a, (iv) bevacizumab and PEG-interferon alfa-2a, and (v) obinutuzumab, for the treatment of locally advanced or metastatic solid tumors (NCT02174172). The aim is to compare on the same trial the results from these different strategies. The rationale is to restore the antitumor immune response by blocking immune checkpoints, while inducing cell cycle arrest, apoptosis or differentiation, which will led to tumor growth inhibition, concomitant with T cell and NK cell activation, inhibition of angiogenesis and induction of cytokine expression, through the administration of interferon alpha (321).

### Anti-Cytokine Antibodies

On the previous section, the aim was to provide exogenous cytokines to burst the antitumor immune response. Here, the aim is to disrupt the balance of other cytokines such as IL-17 or IL-1, that may hinder the antitumor immune response.

An example, for the treatment of patients with multiple myeloma, uses the anti PD-1 mAb PDR001 in combination with the mAb CJM112 (targeting IL-17) or with the Smac Mimetic LCL161 drug (an IAP inhibitor) (NCT03111992). This strategy aims to restore the cellular immune response inhibiting checkpoint signaling, changing the cytokine balance by decreasing the available IL-17 and favoring tumor cell apoptosis. A more complex clinical trial was used for the treatment of colorectal cancer, triple-negative breast cancer, NSCLC and adenocarcinoma, where the anti-PD-1 mAb PDR001 (13) was used in combination with either (i) the anti-interleukin-1 beta (IL-1b) mAb canakinumab (214), (ii) the anti-IL-17 mAb CJM112 (219), (iii) the small molecule inhibitor trametinib (MAPKK1 and MAPKK2 inhibitor) or (iv) the EGFR antagonist nazartinib (NCT02900664). The aim is to inhibit the immune checkpoint, while either simultaneously suppressing the inflammatory responses (blocking IL-1b or IL-17), or inhibiting tumor cell proliferation with mitogen-activated protein kinase and EGFR inhibitors.

### Antibodies Targeting Angiogenesis

Unlike to what happens with hematologic tumors, the growth of a solid tumor is concomitant with a local increase in nutrient and oxygen consumption and secretion of metabolites, requiring neo-vascularization for its growth. Therefore, some of the antitumor therapies aim to interfere with the neo-vascularization process, either by including antibodies against the soluble ligands, or against their receptors present in the cell surface of endothelial cells (322).

The anti-VEGF-A mAb bevacizumab, able to inhibit angiogenesis is currently being tested in combination with other therapeutic agents to determine its usefulness for cancer treatment. These combinations include: cetuximab, in advanced lung cancer (NCT00368992); MEDI3617 (anti-Ang-2) (238), for advanced solid malignancies (NCT01248949); the mAb drozitumab (PRO95780) (223) against death receptor 5 (DR5/TRAIL-R2), in metastatic colorectal cancer (NCT00851136); NK immunotherapy, in recurrent solid tumors (NCT02857920); and atezolizumab (NCT03038100, NCT02659384), nivolumab (NCT02873962), or pembrolizumab (NCT02853318) for the treatment of ovarian, fallopian tube, or primary peritoneal cancer. Bevacizumab has also been combined with MNRP1685A (242, 323), a mAb against membrane-bound endothelial cell co-receptor neuropilin-1 (NRP1), overexpressed in certain tumor cells, for advanced or metastatic solid tumors (NCT00954642). MNRP1685A prevents angiogenesis by blocking binding of VEGF, VEGF-B, and placental growth factor 2 to neuropilin-1, resulting in vessel immaturity. Other combinations of bevacizumab include parsatuzumab (247), a mAb against the vascular-restricted extracellular matrix protein epidermal growth factor-like domain multiple 7 (EGFL7), upregulated during angiogenesis and overexpressed on the cell surface of different solid tumors, for the treatment of metastatic colorectal cancer (NCT01399684). Parsatuzumab inhibits vascular development regulated by EGFL7, affecting to the survival and migration of endothelial cells during angiogenesis. An additional combination used bevacizumab and anti-VEGFC/Flt4 (VGX-100) (265) for metastatic solid tumors (NCT01514123). The rationale is to simultaneously inhibit vascular and lymphatic endothelial cell proliferation and angiogenesis.

A different approach is to target angiogenesis with the Anti-VEGFR-2 mAb ramucirumab, in combination with the anti-c-MET [hepatocyte growth factor receptor (HGFR)] mAb emibetuzumab (227), on advanced refractary solid tumors (NCT02082210). The rationale of the trial is to inhibit angiogenesis and MET signaling on the tumor cells (227). A similar strategy has been used where instead of using an anti-HGFR mAb, uses ficlatuzumab (229, 324), a mAb against the c-MET ligand (HGF), in combination with cetuximab (NCT02277197).

A similar strategy targets molecules with an expression highly restricted to the vascular endothelium. An example is the combination of pembrolizumab with demcizumab (222), a mAb that blocks the interaction of anti-delta-like ligand 4 (DLL4) with Notch-1 and Notch-4, inhibiting Notch-mediated signaling and gene transcription, impairing the productive growth of new blood vessels (325) (NCT02722954). Pembrolizumab avoids the immunosuppression by immune checkpoint signaling while demicizumab prevents angiogenesis.

In K-ras wild-type metastatic colorectal cancers, cetuximab mAb was used in combination with the alphaVbeta3 (vitronectin receptor) integrin inhibitor EMD 525797, an anti-alphaV integrin subunit mAb (226) (NCT01008475). AlphaVbeta3 integrin is a cell adhesion and signaling receptor expressed on the surface of tumor endothelial cells, with a crucial role in their adhesion and migration. The aim of the trial is to inhibit angiogenesis and endothelial cell interaction(s) with other cells or with the extracellular matrix, required for tumor angiogenesis and metastasis. A similar study in solid tumors combines bevacizumab with intetumumab (235, 326), a pan alpha-v human mAb that blocks both alpha-v beta-3 and alpha-v beta-5 integrins, resulting in inhibition of integrin-mediated tumor angiogenesis and tumor growth (NCT00888043).

The remaining clinical trials on this section, all of them combine an anti-checkpoint antibody (either anti-CTLA-4, PD-1 or PD-L1) with anti-angiogenic antibodies such as the anti-Angiopoietin 2 (Ang-2) mAb (MEDI3617) in metastatic melanoma (NCT02141542); with the antibody carotuximab (TRC105) (215) that recognizes the endothelial cell surface protein endoglin, essential for angiogenesis, in metastatic NSCLC (NCT03181308); with antibodies specific for Ang-1 and Ang-2, which prevent their interaction with their target tie2 receptors (NCT00861419); with vanucizumab (bispecific anti-VEGF/Ang-2 antibody) (263), in advanced or metastatic solid tumors (NCT01688206). The bispecific mAb targets both VEGF-A and Ang-2, which are upregulated in a variety of tumor cell types, play key roles in tumor cell proliferation, angiogenesis and metastasis. The anti-VEGF-A arm is based on bevacizumab and the anti-Ang-2 arm is based on the anti-Ang-2 antibody LC06 (263). It simultaneously binds and neutralizes both VEGF-A and Ang-2. This prevents the activation of both VEGF-A/VEGFR- and Ang-2/Tie2-mediated signaling pathways, resulting in the inhibition of proliferation of VEGF-A- and/or Ang-2-overexpressing tumor cells (263, 327).

Another strategy is to combine the anti-EGFR mAb panitumumab with an anti-hepatocyte growth factor mAb rilotumumab (252) or ganitumab (230), an anti-insulin-like growth factor 1 receptor (IGF-1R) mAb in metastatic colorectal cancer with wild-type KRAS (NCT00788957) (328). The rationale here is to simultaneously inhibit strong proliferative signals triggered by EGFR and c-MET.

### Antibodies Combined with Effector Cells

In some patients, the number of cells from the innate or adaptive immune system could be decreased by the effects of previous treatments. In these cases, treatments with antibodies, whose mechanisms of action depend on immune system cell effector functions (i.e., ADCC, ADCP, etc.), could be compromised. In these cases, either autologous (harvested prior to the treatment) or allogeneic cells (NK cells, T cells, CTL cells, dendritic cells (DC), etc.) can be administered concomitantly with the therapeutic antibodies.

A combination of mAb and cells for the treatment of hematological malignancies combines the anti-CD22 mAb epratuzumab (228) with haploidentical NK cells and low-dose exogenous IL-2, for the treatment of relapsed acute lymphoblastic leukemia (NCT00941928). CD22 is a cell surface glycoprotein present on mature B cells and on many B cell malignancies. In this example, since epratuzumab action involves ADDC, the exogenous administered haploidentical NK cells strengthens its effects. The exogenous IL-2 induces NK cell proliferation, activates cytotoxic immune responses against the tumor and induces expression of certain cytotoxic cytokines, such as interferon-gamma (IFNgamma) and transforming growth factor-beta. In another example, NK cells were used in combination with nivolumab for the treatment of recurrent solid tumors (NCT02843204), strengthening the endogenous immune response with the anti-checkpoint antibodies and increasing the NK cell load. Another combination used for the treatment of recurrent solid tumors uses NK cells in combination with bevacizumab (NCT02857920), increasing the NK cell load and simultaneously targeting tumor neo-vascularization. The last combinations to be mentioned with effector cells use pembrolizumab, administrated with autologous dendritic cells-cytokine induced killer cell (DC-CIK), for advanced solid tumors (NCT03190811), or the anti-PD-1 mAb, that was used *in vitro* to activate and expand DC-CIK from the patient’s peripheral blood, before infusion (NCT02886897). These clinical trials aim to target the immune checkpoint and increasing the load of cytolytic cells with the DC-CIK.

### Bispecific Antibodies

Nowadays the FDA and EMA allow clinical trials where the therapeutic agent is a combination of two antibodies. Bispecific antibodies may be considered as a particular combination where both antibodies are in a single molecule. This type of antibodies allows to put in close proximity the tumor cell with an effector cell, a cytokine, etc., or to re-direct the immune response of cytotoxic T cells bypassing antigen recognition through the TCR (329).

On all the trials on hematological tumors using bispecific mAb reported here, the same bispecific mAb blinatumomab (biespecific CD19-CD3) was used in different combinations. An example combines blinatumomab with the anti-CD20 mAb rituximab, for non-receptor tyrosine kinase (ABL)-negative B lineage acute lymphoblastic leukemia (NCT02003222). The rationale is to target the CD20^+^ B cells, while putting in close contact T cells (CD3^+^) with CD19^+^ B cells, to mount a strong cytotoxic T cell response. The rest of the trials from this group, all of them combine mAb targeting immune-checkpoints (CTLA-4, PD-1, or PD-L1) with the bispecific antibodies. These include blinatumomab in relapsed or refractory precursor B-lymphoblastic leukemia (NCT02879695) or for relapsed or refractory B cell acute lymphoblastic leukemia (NCT03160079). The rationale for these trials is to avoid the suppression of antitumor responses, while putting in close contact T cells (CD3^+^) with CD19^+^ B cells.

Bispecific antibodies in combination have also been used for the treatment of solid tumors. For most of the examples, one of the arms of the bispecific mAb identifies a tumor-associated antigen, such as the bispecific antibody RO6958688 (bispecific CD3-CEA) combined with atezolizumab, for the treatment of advanced and metastatic solid tumors (NCT02650713). The bispecific antibody RO6958688 (253, 254) recognizes, on the one side, the CD3 molecule of the TCR and, on the other side, the CEA, an antigen overexpressed in several tumors. The rationale of the trial is to block the binding of PD-L1 to its receptor avoiding the suppression of antitumor responses, while putting in close contact T cells (CD3^+^) with the CEA^+^ tumor cells, inducing a strong T cell activation which may result in a potent antitumor CTL response. Similarly, solid tumors were treated with atezolizumab in combination with bevacizumab or with vanucizumab, a bispecific mAb that simultaneously targets VEGF and Ang-2 (NCT02715531). This treatment aims to block the immune checkpoint suppression while inhibiting angiogenesis.

Another example of targeting tumor-associated antigens is the use of *in vitro*-activated T cells armed with GD2Bi-aATC, a bispecific antibody that recognizes CD3 and GD2 (231, 232), in combination with IL-2 and GM-CSF in patients with neuroblastoma or osteosarcoma (NCT02173093). The rationale of this study is to generate *in vitro* activated T cells that are infused in the patient after binding to the bispecific mAb, which will direct them to the tumor, generating a potent CTL response to kill tumor cells. Exogenous IL-2 and GM-CSF are added to maintain these cells and generate an inflammatory environment surrounding the tumor. In addition, another approach that has been used is to combine the anti-HER2 antibody trastuzumab with a bispecific MM-11 mAb anti-ErbB2/anti-ErbB3 mAb (241) for the treatment of HER2^+^ breast cancer (NCT01097460). The aim is to simultaneously inhibit signaling through this family of tyrosine kinase receptors.

Another approach is to use bispecific mAb to inhibit angiogenesis in solid tumors. The two examples we describe use the bispecific mAb vanucizumab (VEGF-A/Ang-2). In one of them, it is used in combination with RO7009789 (255), an antibody with immunostimulatory effects that recognizes CD40, a member of the TNF receptor superfamily, for the treatment of metastatic solid tumors (NCT02665416). In the other example, it is used either alone or in combination with atezolizumab (PD-L1) in advanced or metastatic solid tumors (NCT01688206). The rationale for both cases is to either use an anti-CD40 agonist or an anti-checkpoint antibody to burst the antitumor immune response, while inhibiting simultaneously angiogenesis by blocking the VEGF-A/VEGFR- and Ang-2/Tie2-signaling pathways.

### Antibodies with Immunomodulatory Effects

In a clinical trial for metastatic colorectal cancer, imalumab (BAX69) (233), a mAb that identifies MIF is used in combination with panitumumab (NCT02448810). BAX69 abrogates MIF signaling and MIF-mediated secretion of cytokines (IL-1β, TNF-α, etc.) and inhibits proliferation of MIF overexpressing tumor cells, together with the antiproliferative effects of panitumumab (anti-EGFR mAb).

Other examples, used for solid tumors, combine nivolumab with cabiralizumab, an anti-colony-stimulating factor 1 receptor (CSF1R) mAb, which inhibits binding of its ligands (CSF-1 and IL-34), blocking the production of inflammatory mediators by macrophages and monocytes and preventing osteoclast activation (NCT02526017; NCT03158272). The aim is to inhibit the tumor-induced immune suppression with nivolumab, and to block with cabiralizumab the recruitment of CSF1R-dependent tumor-associated macrophages (TAMs). Cabiralizumab also enhances T cell infiltration and antitumor T cell immune responses.

Another immunomodulatory antibody, anti-CD73 (BMS-986179) (209–211), has been used in combination with nivolumab on advanced or spread solid cancers (NCT02754141). In this case, the use of an antibody against the cell surface enzyme CD73 turns out to be very interesting. CD73 is overexpressed in many tumors and catalyzes the conversion of extracellular nucleotides into nucleosides, generating adenosine (211). The anti-CD73 antibody prevents the conversion of AMP to adenosine, which releases the inhibition of T cell, DC, and NK activities, induces the activation of macrophages, and reduces the activity of both myeloid-derived suppressor cells and regulatory T cells (209–211). This treatment was designed to abrogate the immunosuppressor effects of both, the immune checkpoint with nivolumab and the metabolic checkpoint with BMS-986179.

In addition to antibodies that release the inhibitory effects of immune-checkpoints, there are other antibodies that are able to directly activate the immune response. The following examples represent clinical trials where these immunostimulatory antibodies are used. One of them combines avelumab, which suppresses the signaling through negative immune checkpoints, with either the anti-cytokine antibody PD-0360324 (anti-CSF-1 mAb) (248); or with the immunostimulatory antibodies PF-04518600 (anti-OX40 mAb), or utomilumab (an anti-CD137 mAb) (262) (NCT02554812); whereas another combines the anti-OX40 mAb (MOXR0916) with atezolizumab in locally advanced or metastatic solid tumors. (NCT02410512). The aim is to inhibit the PD-1/PD-L1 axis (avelumab or atezolizumab), simultaneously bursting the immune response through OX-40, CD137, or blocking TAMs generation with the anti-CFS-1 (Aspelagh, 2016). A similar strategy uses atezolizumab in combination with varlilumab (an agonistic anti-CD27 mAb), which results in an increase of the CTL response against CD27 ligands expressed on tumor cells (NCT02543645).

The anti-CD40 mAb RO7009789 activates and triggers proliferation of antigen-presenting cells (APC) and activates B and T cells, resulting in an enhanced immune response. When CD40 is expressed in solid tumor cells, RO7009789 leads to apoptosis and decreased tumor growth. This antibody in combination with the CSF1R inhibitory antibody emactuzumab has been used for the treatment of advanced solid tumors (225) (NCT02760797). Related examples combine anti-CD40 mAb either with nivolumab for the treatment of metastatic pancreatic adenocarcinoma (NCT03214250) or with the bispecific antibody vanucizumab (anti-VEGF-A and anti-Ang-2) (NCT02665416). The rationale is to activate the immune response through CD40 while inhibiting the angiogenesis blocking the binding of VEGF-A and Ang-2 to their receptors.

### Adjuvants and other Immunostimulatory Agents

Another possible strategy is to combine therapeutic antibodies with molecules carrying repeated structural motifs that cannot be synthesized by vertebrates, and bind to pattern recognition receptors present in cells from the innate arm of the immune system (i.e., beta glucan that binds the C-type lectin receptor Dectin-1). These molecules are able to regulate the threshold of the immune response as an adjuvant, changing the secreted cytokine expression pattern. A particular example of this type of agents is the use of an attenuated preparation of the BCG (Bacille Calmette–Guerin) strain of *Mycobacterium bovis*, with potential immunostimulatory activity for the treatment of patients with bladder cancer (330).

An example, combining mAb and adjuvants is the use of BTH1704 (212), an mAb against MUC1, an aberrantly glycosylated antigen overexpressed on the surface of a variety of cancer cells, in combination with a polysaccharide beta 1,3/1,6 glucan derived from the cell wall of *Saccharomyces cerevisiae* (PGG Beta-Glucan), for the treatment of patients with advanced pancreatic cancer (NCT02132403). The rationale of this trial is to directly target the tumor with the anti-MUC1 mAb, while unspecifically stimulate the immune response with beta glucan by binding to an alternate site on the neutrophil complement receptor 3 (CR3), priming the neutrophil to become cytotoxic after binding to complement on tumor cells *via* CR3. In addition, this agent may induce hematopoietic progenitor cell mobilization.

### Antibodies in Combination with Vaccines

Tumor cells carry antigens which can be recognized as non-self by the immune system. In some cases, however, the microenvironment in which these tumor antigens are presented do not allow to evoke an immune response. There is a plethora of possibilities to burst the antitumor immune response, one of them is to use tumor antigens as a vaccine. In the context of antitumor therapies, anti-idiotipic antibodies represent a particular type of vaccines.

Since the interaction of antibodies with other molecules is based on structural complementarity, antibodies that recognize the region of an antibody that interacts with its antigen (anti-idiotipic antibodies) might mimic the structure of this antigen (i.e., a tumor marker). Thus, the anti-idiotipic antibodies might act as an antitumor vaccine able to trigger a host immune response to kill tumor cells. Combining chemotherapy and radiation therapy with vaccine therapy may help to kill tumor cells more effectively.

An example of anti-idiotipic antibodies used as vaccines in antitumor therapy is abagovomab, an IgG1 anti-idiotype mAb, that functionally mimics the 3D structure of a specific epitope on the ovarian cancer tumor-associated antigen CA-125 (203). Its variable region acts as a surrogate antigen for CA-125, bestowing potential antineoplastic activity; it has been used in ovarian epithelial, fallopian tube, or peritoneal cancer (NCT00058435). Another example of vaccine therapy combines two anti-idiotipic antibodies, 11D10 (201) an mAb that mimics an epitope of the high molecular weight human milk fat globule glycoprotein, expressed at high levels by human breast and other tumor cells and 3H1 an mAb that mimics an epitope of the tumor-associated protein CEA (199). This combination has been used for the treatment of colorectal cancer metastatic to the liver (NCT00033748). The 11D10 mAb has also been used in other combinations, for example, with a GD2 anti-idiotype mAb vaccine, together with chemotherapy and radiotherapy, for the treatment of limited-stage SCLC (NCT00045617).

A study combining the anti-idiotipic mAb abagovomab, which mimics a specific epitope on CA-125 with stereotactic body radiation therapy (SBRT), chemotherapy, and the synthetic antiviral agent nelfinavir mesylate, which selectively binds to and inhibits human immunodeficiency virus protease, has been used for the treatment of locally advanced pancreatic cancer (NCT01959672).

Currently, other types of vaccines are being used with mAb in combination with agents that allow to evoke an immune response against tumor antigens. The mAb in these combinations may strengthen the evoked immune response. For the examples described below, the combination contains antibodies that either disrupt the PD-1/PDL-1 axis or that block the binding of B7-1 and B7-2 to CTLA-4 allowing T cell co-stimulatory signals and activation, unless otherwise specified. One example is the treatment with a peptide from Wilms tumor 1 antigen on recurrent ovarian cancer (NCT02737787). Another, more complex example is to administrate the peptide vaccine PVX-410 (derived from X-box-binding protein 1-unspliced XBP1-US, XBP1-spliced syndecan-1, and CS1), to treat triple-negative breast cancer tumors (NCT02826434). A third example uses a HER2 intracellular domain peptide in combination with the polysaccharide-K as adjuvant, in HER2^+^ recurrent breast cancer patients, which are receiving pertuzumab or trastuzumab (NCT01922921). The rationale for this trial is to combine the effects of the anti-HER2 mAb with using a HER2 peptide to switch the B and T cell responses through APC activation. Otherwise, peptides could be presented as a fusion protein, such as in CIMAvax vaccine (EGF-rP64K/Montanide ISA 51), which triggers a strong humoral immune response against EGF and has been used in NSCLC (NCT02955290). There are also personalized neoantigen cancer vaccines, such as NeoVax for the treatment of high-risk renal cell carcinoma (NCT02950766).

Another approach to generate vaccines is to use modified virus such as Ad-CEA vaccine, an oncolytic adenovirus encoding an epitope of human CEA (331), used for the treatment of patients with previously untreated metastatic colorectal cancer (NCT03050814). This vaccine may induce both humoral and cellular immune responses against CEA^+^ tumor cells.

A different example uses the CV301 (CEA-MUC-1-TRICOM Vaccine) viral vaccine, which contains a version of the recombinant vaccinia viral vector and a recombinant fowlpox viral vector encoding both CEA and MUC-1, in combination with TRICOM [co-stimulatory molecules, B7-1, intracellular adhesion molecule 1 (ICAM-1), and LFA-3] (332). It may enhance presentation of CEA and MUC-1 to APC and subsequently a CTL response against the tumor cells, it has been used in previously treated NSCLC (NCT02840994). A similar approach has been evaluated for the treatment of prostate cancer using a recombinant vaccinia virus encoding a modified peptide of the prostate-specific antigen and TRICOM (NCT00113984). This viral vaccine may enhance antigen presentation and may activate a CTL response.

Attenuated bacteria have also been used as carriers for antitumor vaccines. For example, the attenuated Listeria ADXS11-001 encoding a papillomavirus type 16 E7 fused to a non-hemolytic listeriolysin O protein, it has been used for the treatment of cervical and Head and Neck Cancer (HNSCC) (NCT02291055). The rationale is to mount a CTL response against cancer cells overexpressing the cell surface glycoprotein HPV 16 E7, overexpressed in the majority of cervical cancer cells.

More sophisticated approaches use tumor vaccines, such as GM.CD40L, which is a cell-based vaccine composed of irradiated tumor cells transduced with GM-CSF and CD40-ligand (CD40L) genes (333). Upon administration, this vaccine may stimulate an antitumoral DC-mediated immune response, it has been used in lung adenocarcinomas (NCT02466568). Another example that does not use anti-checkpoint antibodies but combines the anti-HER2 mAb trastuzumab with a cell-based vaccine, consisting of two irradiated allogeneic mammary carcinoma cell lines genetically modified to secrete human GM-CSF, has been used for the treatment of HER2^+^ metastatic breast tumors (NCT00399529). An additional example of cell-based vaccines uses GVAX (334), a pancreatic cancer vaccine and IMC-CS4, a macrophage targeting mAb (CSF1R inhibitor) for the treatment of pancreatic adenocarcinomas (234). GVAX is composed of irradiated, whole tumor cells (autologous or allogeneic), genetically modified to secrete GM-CSF (NCT03153410).

The immune response against the tumor can be busted using also DNA, RNA, or liposome-based vaccines. As an example, triple-negative breast cancers have been treated with neoantigen DNA vaccine combined with anti-immune-checkpoint antibodies (NCT03199040). Other vaccines use autologous DC loaded *in vitro* with Cytomegalovirus pp65-lysosomal-associated membrane protein mRNA as a vaccine, in combination with the anti-IL2R alpha mAb basiliximab (207), in glioblastoma multiform (NCT00626483). The rationale is to restore the number of immunosuppressive T regulatory cells during recovery from therapeutic temozolomide-induced lymphopenia, together with a synergistic enhancement of vaccine-driven CTL responses. Another study describes the use of the immuno-modulating mAb varlilumab (anti-CD27, TNFR family) in combination with a liposome-based vaccine consisting of two peptides from MUC1 and the toll-like receptor 4 encapsulated in liposomes (NCT02270372). This immunization stimulates both cellular and humoral responses.

Another clinical trial uses trastuzumab and an allogeneic GM-CSF-secreting whole cell breast cancer vaccine for HER-2^+^ breast tumors. This study will also test whether cyclophosphamide can eliminate the suppressive influence of regulatory T cells. The vaccine consists of two irradiated allogeneic mammary carcinoma cell lines genetically modified to secrete human GM-CSF (NCT00399529).

Pidilizumab (anti-PD-1) (250) in combination with a DC fusion vaccine, following autologous stem cell transplantation has been used on multiple myeloma (NCT01067287). The rationale is to disrupt the PD-1/PD-L1 axis, while vaccinating the patients with de DC fusion vaccine, which consists of DC fused to the patient’s myeloma, where the myeloma antigens will be presented by HLA class I to CD8^+^ T cells, allowing their activation and mounting a CTL response. This is done on patients after an autologous transplantation with HSCs. A similar approach uses nivolumab vaccine with autologous DCs pulsed with tumor lysate antigen, in patients with recurrent glioblastoma (NCT03014804).

Another study combines lymphodepletion with anti-CD45 mAb with a vaccine generated from autologous DC and Epstein Barr virus (EBV)-infected lymphoblastoid cell lines transduced with an LMP1/LMP2-expressing adenoviral vector, which are irradiated, and then used to stimulate and expand autologous CTL to produce LMP1-/LMP2-specific CTL *ex vivo*, for the treatment of EBV^+^-nasopharyngeal carcinoma (NCT00515957). The rationale of this trial is to deplete autologous CD45^+^ cells, then generate a cell-based vaccine to activate *ex vivo* specific CTL that are then infused into the patient.

### Other Strategies

In some approaches, a fusion protein between the antibody and a tumor antigen is used in combination with other therapies. For example, on the treatment of NY-ESO 1^+^ NSCLC, where atezolizumab was combined with both the adjuvant poly-ICLC (a synthetic complex of carboxymethylcellulose, polyinosinic-polycytidylic acid and poly-L-lysine double-stranded RNA) and DEC-205/NY-ESO-1 [CDX-1401, a fusion protein between a mAb directed against the endocytic DC receptor DEC-205, linked to the tumor-associated antigen (NY-ESO-1)] (NCT02495636) (217). Atezolizumab will inhibit immune checkpoints negative signals, while the internalization by DC of the mAb–antigen fusion protein may specifically deliver the NY-ESO-1 molecule and trigger a CTL response against cancer cells expressing this antigen. Simultaneously, the adjuvant may stimulate the release of cytotoxic cytokines by inducing IFNgamma production. A similar approach, for the treatment of melanoma patients, using CDX-1401 combined with a neoantigen-based melanoma-poly-ICLC vaccine and a recombinant Flt3 Ligand (CDX-301) (NCT02129075). This treatment should boost the immune system to mount a CTL response against cancer cells expressing NY-ESO-1. In addition, the adjuvant may induce IFNgamma production and the recombinant Flt3 ligand may stimulate the proliferation and mobilization of bone marrow precursor cells, including CD34^+^ cells, and DCs.

An additional strategy would be to use scavengers of the ligand with low immunogenicity. For example, sEphB4-HAS, a human serum albumin (HAS) fused with the extracellular domain of tyrosine kinase ephrin type-B receptor 4 (sEphB4) is combined with pembrolizumab, for the treatment of NSCLC or HNSCC (NCT03049618). Pembrolizumab inhibits negative immune checkpoint signals, whereas EphB4-HSA is expected to decrease angiogenesis and cell growth of Efnb2 and/or EphB4 overexpressing tumor cells, while the albumin moiety will avoid renal clearance of the fusion protein, increasing its half-life without affecting immunogenicity.

Another strategy would be to target matrix enzymes required for tumor invasiveness. An example is the use of nivolumab combined with andecaliximab (GS-5745) (204, 205), an inhibitory mAb of matrix metalloproteinase 9 (MMP-9) in recurrent gastric or gastroesophageal junction adenocarcinomas (NCT02864381). Since MMP-9 activity is associated with tumor invasion and metastasis (335), the rationale is that andecaliximab will inhibit extracellular matrix protein degradation and angiogenesis, while nivolumab will interfere with the PD-1/PD-L1 axis.

Pembrolizumab (PD-1) was used in combination with CVA21 (CAVATAK™), coxsackievirus A21, a naturally occurring enterovirus with potential antitumor activity. This combination was used for advanced NSCLC (NCT02824965). CVA21, intra-tumor administered, targets and binds the ICAM-1 and decay acceleration factor, cell surface molecules, both overexpressed on certain malignant cells (336, 337). After entering the cells, the virus replicates causing cell lysis. This, together with the inhibition of the immune checkpoint, results in a reduction of tumor cell growth.

Another strategy combines conatumumab (220), an agonist mAb directed against the extracellular domain of human tumor necrosis factor-related apoptosis-inducing ligand (TRAIL) receptor 2 (TRAIL-R2), also known as DR5, with the anti-IGF-1R mAb ganitumab, in patients with advanced solid tumors without disease progression whose previous studies were closed (NCT01327612). TRAIL-2 and IGF-1R are expressed by a variety of solid tumors and cancers of hematopoietic origin. Conatumumab mimics TRAIL activity, activating caspase cascades and inducing tumor cell apoptosis, while ganitumab inhibits IGF-1 binding and, therefore, the PI3K/Akt pathway. This treatment may result in the inhibition of tumor cell proliferation and the induction of tumor cell apoptosis. Another clinical trial combining tigatuzumab, a mAb targeting the death receptor TRAIL-R2 with abraxane, an albumin-stabilized nanoparticle containing paclitaxel, non-covalently coated with the anti-CD20 mAb rituximab, in patients with metastatic, triple-negative breast cancer (NCT01307891). The relevance of the trial is that combines an anti-TRAIL-R2 mAb that induces death, while albumin stabilizes the complex, whereas rituximab allows to target paclitaxel to CD20^+^ cells, minimizing toxicity on normal cells. A strategy being used on EGFR^+^ tumors is to combine an anti-immune checkpoint antibody with the anti-EGFR mAb necitumumab (NCT02451930) or nimotuzumab (245) (NCT02947386). Or the same strategy, but using B-701, a neutralizing mAb directed against the fibroblast growth factor receptor type 3, in combination with atezolizumab in urothelial cell carcinoma (NCT03123055).

One of the most sophisticated clinical trials combines chemotherapy, bevacizumab, avelumab, ALT-803 (IL-15 super agonist), aNK (allogenic human NK-92 cell line, expressing CD16 and IL-2), and GI-4000 (a heat-killed recombinant *Saccharomyces cerevisiae* yeast transfected with mutated forms of Ras) with the NANT pancreatic cancer vaccine (ETBX-011) containing a replication-defective adenoviral vector encoding a CEA epitope Ad5-CEA(6D), used for pancreatic cancer (NCT03136406). A clinical trial for colorectal cancer uses a similar combination of chemotherapy, nivolumab, avelumab bevacizumab, cetuximab, SBRT, haNK, ALT-803, and a cocktail of vaccines: ETBX-011, ETBX-021, ETBX-051, ETBX-061, GI-4000, GI-6207, and GI-6301 (NCT03169777). The rationale for these two clinical trials is to hit the tumor simultaneously with a wide spectrum of the available tools against the tumor, where the concentration of each one of the agents can be decreased to minimize the unwanted effects on normal cells.

## Questions and Queries Raised by the Combinations

We hope that we have been able to depict up to here, the huge complexity inherent to the use of therapeutic antibodies in combination with other biological agents for the treatment of cancer. Since most of the clinical trials described in this review are relatively recent (started during the last 7 years), many of them lack results in public databases, including the clinical trials database from NCI, or as published scientific manuscripts. This burst of clinical trials using antibodies in combinations with other biologicals is based on the positive results found by some combinations (anti-HER2 or anti-GD2 mAb), although the complexity increase in these combinations also implies an increase in the possibility of adverse side effects/increased toxicity or lack of additive or synergistic effects of the therapeutic agents. Initially, the therapeutic doses used for combinations were taken from the monotherapeutic trials, although in many cases, the non-toxic concentrations used in monotherapy, turn to be toxic in combinations, generating new toxicity profiles (338). This is of particular relevance when antibodies able to burst the antitumor immune response are used (either to inhibit the immune checkpoint proteins, to block inhibitory NK receptors or to trigger NK cells through activating receptors, etc.), which might lead to a dis-regulation of the immune response, an uncontrolled inflammatory response, and autoimmunity. This problem can also be related to an apparent lack of additive or synergistic effects of the therapeutic agents, where the potential clinical benefits of the combination could be overlooked by the initial toxicity of the mixture. Dose and schedule changes, however, can overcome the toxicity effects, allowing to demonstrate the enhanced clinical benefits of a particular combination (339). The use of CTLA-4 and PD-1 inhibitor antibodies in combination (nivolumab and ipilimumab), improved the treatment efficacy in advanced melanoma, as compared to monotherapies (340). Indeed, this combination has been approved in 2016 by the US FDA for the treatment of metastatic melanoma (341), despite the higher frequency and severity of adverse reactions of the combination, as compared to the corresponding monotherapies (340, 342–344).

It is interesting to note that a combination of TRC105 (carotuximab, anti-endoglin antibody) with bevacizumab was used on a clinical trial for the treatment of patients with advanced cancer, where the combination was well tolerated and clinical activity was observed in a VEGF inhibitor-refractory population (NCT01332721) (345), the same combination failed to improve progression-free survival on patients with refractory metastatic renal cell cancer (NCT01727089) (346). These data clearly suggest that the problem does not strictly lie with the antibody combination, but rather it might be related to the tumor microenvironment, tumor type, the therapeutic approach used, or the clinical history of the patient.

Several examples of clinical trials where antibodies that have been used in combination with other biologicals, which were well tolerated and showed additive or synergistic therapeutic responses have been selected. These include a clinical trial for melanoma patients with low tumor infiltrating lymphocytes, an anti-PD-1 non-responsive phenotype. The combination of pembrolizumab with an intratumoral electroporation of a plasmid coding for interleukin 12 cDNA (pIL-12) showed a 40% clinical response with associated positive immune-based biomarker data and a safety profile (NCT02493361) (347), where the combination of pembrolizumab with the plasmid pIL-12 renders half of the patients responsive to the anti-PD-1. Another example that combines mAb with cytokines is a study of immune activation and antitumor activity in renal cancer of PEGylated human IL-10 (AM0010) in combination with pembrolizumab or nivolumab, the combinations were well tolerated, and CD8^+^ T cell activation was detected (NCT02009449) (348).

Other examples include a phase Ib study, otlertuzumab (TRU-016, an anti-CD37 mAb) in combination with rituximab and bendamustine, which was well tolerated and induced therapeutic responses in the majority of patients with relapsed indolent B-non-Hodgkin lymphoma (NCT01317901) (349). Similarly, the combination of pidilizumab plus rituximab was well tolerated and therapeutically active in patients with relapsed follicular lymphoma (350).

On a phase Ib study of utomilumab (PF-05082566, a 4-1BB/CD137 agonist), in combination with pembrolizumab (MK-3475) in patients with advanced solid tumors had a confirmed complete or partial response in 26.1% of them. Pharmacokinetics and immunogenicity of both mAb were similar when administered alone or in combination. A trend toward higher levels of activated memory/effector peripheral blood CD8^+^ T cells was observed in responders versus non-responders, supporting further investigation of this combination (NCT02179918) (262).

Other combinations include the anti-checkpoint antibody nivolumab in combination with an antibody that blocks the KIR inhibitory receptors in NK cells. On a phase I/II study of the NK-targeted antibody lirilumab (a fully human mAb that blocks inhibitory KIRs on NK cells) in combination with nivolumab in advanced HNSCC demonstrated, in preliminary results, clinical benefit, with deep and durable responses in some patients. This combination demonstrated a manageable safety profile similar to that observed with nivolumab monotherapy (NCT01714739) (347).

Another combination that might be interesting for the future of the field is the combination of oncolytic virus with anti-checkpoint antibodies. Indeed, preliminary results from several clinical trials using the oncolytic virus coxsackievirus A21 (CVA21, CAVATAK) in combination with ipilimumab (NCT01636882) (351) or pembrolizumab (NCT02043665) (348), for the treatment of patients with advanced cancer, showed that these combinations were generally well tolerated and induced antitumor activity. A phase II trial using intratumoral injection of the HF10 oncolytic virus, an attenuated, replication-competent mutant strain of herpes simplex virus type 1, and ipilimumab in patients with unresectable or metastatic melanoma showed therapeutic activity and the treatment was well tolerated (NCT02272855) (352).

For other clinical trials, on early phases, the combination is well tolerated, such as a dose escalation study of the OX40 agonist MOXR0916 and atezolizumab (anti-PD-L1 mAb) in patients with advanced solid tumors, using each agent at its recommended monotherapy dose, was well tolerated. (NCT02410512) (353).

Other clinical trials, including anti-checkpoint antibodies detect clear tumor regression, although with a toxicity higher than reasonable. This is the case for a clinical trial were BMS-986016 (anti-LAG-3 mAb) in combination with nivolumab was administered to patients with hematologic and solid malignancies. Preliminary results demonstrated objective tumor regressions, concomitant with the toxicity characteristic of immune checkpoint blockers (NCT02061761, NCT01968109) (348).

On another group of clinical trials, the main characteristic is that although they are well tolerated in general, they failed to provide significant additive/synergistic therapeutic effects. This is the case of a clinical trial where urelumab (a CD137 agonist), in combination with nivolumab was used for the treatment of hematologic and solid tumor malignancies. This combination did not provide significant additive/synergistic clinical benefits at the doses evaluated (NCT01471210, NCT02253992) (351). In another, urelumab in combination with rituximab or cetuximab was used in patients with refractory lymphoma or selected advanced solid tumors. Although the combinations were safe and well tolerated, with minimal evidence of liver toxicity, they did not demonstrate substantial enhancement of clinical responses or lead to intratumoral immune modulation in these tumor settings (NCT01775631, NCT02110082) (348).

Finally, there are a couple of selected clinical trials using combinations of antibodies and other biologicals that were toxic or had to be terminated on overall benefit–risk assessment. These include a clinical trial with patients with advanced solid tumors, which were treated with a combination of MDX-447 [a bispecific mAb directed to FcγRI (CD64) and EGFR] with G-CSF, although the bispecific mAb alone was well tolerated, the combination was not well tolerated and precluded meaningful dose escalation on a phase I clinical trial (354). A second example is a phase II study of imalumab [BAX69, an anti-oxidized macrophage MIF (oxMIF)] and 5-FU/Leucovorin or Panitumumab (anti-EGRF mAb), versus the standard of care in metastatic colorectal cancer patients, which was terminated (February, 2017) based on overall benefit–risk assessment (NCT02448810), although it was initially reported that this combination was generally safe and well tolerated (355).

The problems that arose with using therapeutic antibodies in combinations has led the Society for Immunotherapy of Cancer to name a Combination Immunotherapy Task Force to identify and prioritize the most promising prospects for combinatorial approaches as well as to address the challenges associated with developing these strategies (339). Furthermore, it seems clear by now, that an improved understanding of pharmacodynamic effects of each agent within a combination will support the rational development of immune-based combinations for cancer treatment (356).

## Conclusion

The broad variety of clinical trials summarized here presents the overwhelming complexity of the use of antibody in combinations for cancer treatments. The antibodies used either interfere with a ligand–receptor interaction, blocking a signaling pathway relevant for tumor growth, or identify tumor-associated antigens, where they somehow induce the death of the tumor cells by ADCC, CDC, and ADCP or directly inducing apoptosis. If the antibodies by themselves cannot kill the tumor cells, they can be conjugated to cytotoxic drugs to exert this function, or directly coupled to radio-labeled agents, where they trigger radiolysis. These antibodies can be combined with other antibodies that rather than directed against the tumor cells, identify targets on the tumor environment. These include antibodies that inhibit tumor-induced vascularization, or antibodies against cells or molecules involved in the immune response. A turning point was the use of mAb that block PD-L1/PD-1 interaction, or anti-CTLA-4 mAb; all of them disrupting the tumor-induced suppression of antitumor immune responses. In addition, mAb are used in combination with vaccines with the aim of evoking an antitumor response, either against a single tumor antigen or against a broad spectrum of antigens, for example when using irradiated tumor cells expressing pro-inflammatory cytokines.

It is obvious that there are many challenges to be solved regarding antibodies in combinations as antitumor therapeutic agents. One of the most relevant challenges is the possible increase of toxicity of the combinations, concomitant with an increased complexity of the trials. This has to be carefully evaluated for each combination to identify the conditions giving the highest efficacy/toxicity ratio. This is of particular interest when the aim is to burst the antitumor host immune response, where a small response could fail to kill the tumor, but an over-response could lead to unwanted inflammatory or autoimmune processes. Another relevant challenge to be solved is the identification of new/additional biomarkers that would allow a personalized follow-up of the patient’s status, which would be required due to the tumor and the patient’s genotypic and phenotypic differences.

The current clinical trials suggest that in the future anti-cancer therapies will combine antibodies that block signaling cascades, or identify tumor-associated antigens with others that disrupt the tumor-induced immuno-suppression, together with vaccines that evoke an antitumor immune response, activated effector cells or CAR T cells. These combinations could also contain anti-cytokine antibodies, or cytokines, to burst the immune response. The aim will be to directly target tumor antigens or signal pathways, and at the same time interfere with the “immune history” of the organism, to make the patient’s own body aware of the tumor presence and simultaneously help it to make a strong antitumor response. Thus, it will be the patient’s immune response against the tumor the ultimate mechanism responsible for the cure of cancer.

## Author Contributions

All authors contributed to drafting, revising, and approving the final article.

## Conflict of Interest Statement

The authors declare that the research was conducted in the absence of any commercial or financial relationships that could be construed as a potential conflict of interest.

## Appendix

### Key Concepts

Antibody-dependent cell cytotoxicity (ADCC): the binding of an antibody to a cell surface antigen promotes the interaction of the natural killer cell with the Fc antibody fragment and triggers its cytotoxic response.

Adaptive immune response: its main function is to eliminate pathogens and fight cancer. Specifically recognizes antigen though receptors on the surface of T and B lymphocytes. It creates immunological memory, after the initial response to a given antigen, leads to an exacerbated response to subsequent encounters with the same antigen. This is the basis of vaccination.

Antibody-dependent phagocytosis (ADCP): the binding of an antibody to a cell surface antigen allows the opsonization of the cell and promotes its phagocytosis.

Combined therapy: therapy that combines different therapeutic approaches (i.e., chemotherapy, radiotherapy, small molecule drugs, vaccines, antibodies, etc.).

Complement-dependent cytotoxicity (CDC): the binding of an antibody to a cell surface antigen activates the complement cascade, resulting in the cell’s death.

Immune checkpoint: there are checkpoints that receive negative signals from the tumor, inhibiting the antitumor immune response. These include the PD-1/PD-L1 axis (PD-1 receptor on the T cells and to PD-L1 on the tumor cells) and CTLA-4 (on T cells). Antibodies against these molecules (nivolumab, pembrolizumab, atezolizumab, durvalumab, avelumab, and ipilimumab) are able to inhibit these negative signals and promote the antitumor immune response.

Immunotherapy: therapeutic treatment that takes advantage of the immune system response.

Innate immune response: provides immediate defense against infection, on a non-specific manner, is not long-lasting and recognizes pathogens through generic receptors.

Therapeutic antibody: an antibody that can be used for therapeutic purposes, which is able to either act as antagonist, guide a drug toward the tumor cell, where it will act, or kill the tumor cell either directly or by activating immune response mechanisms (ADCC, CDC, and ADCP).

Vaccine: compounds inoculated in an organism to evoke a primary immune response, generating immunological memory able to protect against the antigen.
