# Supplementary material for: New Strategies Using Antibody Combinations to Increase Cancer Treatment Effectiveness
Source: Front Immunol. 2017 Dec 21;8:1804. doi: 10.3389/fimmu.2017.01804 (PMC5742572; doi:10.3389/fimmu.2017.01804)
Supplement: Supplementary file 1 [file table_1.PDF]

Corraliza-Gorjón I, Somovilla-Crespo B, Santamaria S, Garcia-Sanz JA and Kremer L (2017) New Strategies Using Antibody Combinations to Increase Cancer Treatment Effectiveness. Front. Immunol. 8:1804. doi: 10.3389/fimmu.2017.01804

Suppl. Table I: Summarized characteristics of selected clinical trials including antibodies in combination with other biologicals for cancer therapy

Clinical trial identifier; Sponsor and collaborators	Official Title; Phase	Study status; First posted; Completion date; Last Update	Condition	Antibody; Other drugs, biologics	Study results
NCT00033748; Alliance for Clinical Trials in Oncology / NCI[1]	Sequential Phase II Study of the Anti-Idiotype Monoclonal Antibody Vaccine CeaVac and TriAb in Patients with Minimal Metastatic Colorectal Cancer; Phase II	Completed; January 2003; June 2010; July, 2016	mCRC	11D10 anti-idiotype, 3H1 Alu Gel	The treatment was well tolerated but did not improve the 2-year RFS when compared with the expected value of 40% reported for hepatic resection alone (1).
NCT00113984; NCI	Phase I Trial of a PSA Based Vaccine and an Anti-CTLA-4 Antibody in Adults with Metastatic Androgen Independent Prostrate Cancer; Phase I	Completed; June 2005; December 2011; October 2017	Prostatic Neoplasms	Ipilimumab; PROSTVAC-V/TRICOM, PROSTVAC-F/TRICOM, Sargramostim	Completed enrolment 30 patients with no dose-limiting toxic effects. The use of a vaccine targeting PSA did not seem to exacerbate the irAEs associated with ipilimumab (2).
NCT00368992; NCI	A Phase II Trial of Combination Carboplatin, Paclitaxel, Cetuximab and Bevacizumab (NSC-704865) Followed by Cetuximab and Bevacizumab in Patients with Advanced Non-Small Cell Lung Cancer; Phase II	Completed; August 2006; October 2010; September 2015	Adenocarcinoma of the Lung, Adenosquamous CLC, Bronchoalveolar CLC, Large CLC, NSCLC, SCLC	Cetuximab, Bevacizumab; Paclitaxel	This was the first reported study to combine carboplatin, paclitaxel, cetuximab and bevacizumab (S0536). It demonstrated that the regimen is safe and efficacious as first-line treatment for advanced NSCLC. Further evaluation of this regimen is in the phase III trial S0819 (3).
NCT00399529; Sidney Kimmel Comprehensive Cancer Center	A Feasibility Study of Combination Therapy with Trastuzumab, Cyclophosphamide, and an Allogeneic GM-CSF-secreting Breast Tumor Vaccine for HER-2/Neu-Overexpressing Metastatic Breast Cancer; Phase II	Completed; November 2006; February 2010; December 2012	Breast Neoplasms	Trastuzumab; Allogeneic GM-CSF-secreting breast cancer vaccine, Cyclophosphamide	This combination immunotherapy was safe, with clinical benefit rates at 6 months (55%) and 1 year (40%). Median PFS and OS durations were 7 months and 42 months respectively. Increased HER2-specific DTH developed in 7 of 20 patients, with a trend toward longer PFS and OS in DTH responders. Polyfunctional HER2-specific $CD8^+$ T cells progressively expanded across vaccination cycles (4).
NCT00515957; Baylor College of Medicine	Administration of LMP1- and LMP2-Specific Cytotoxic T-Lymphocytes Following CD45 Antibody Administration to Patients With EBV-Positive Nasopharyngeal Carcinoma; Phase I	Withdrawn prior to enrollment; August 2007; Not provided; April 16, 2012	Nasopharyngeal carcinoma	Anti-CD45; Genetically modified CTLs	Withdrawn prior to enrollment (Source: www.clinicaltrials.gov).

NCT00788957; Amgen	A Randomized, Phase 1b/2 Trial of AMG 102 or AMG 479 in Combination with Panitumumab Versus Panitumumab Alone in Subject with Wild-Type KRAS Metastatic Colorectal Cancer; Phase I, II	Completed; November 2008; October 2013; July 2015	Colon Cancer, CRC, Gastrointestinal Cancer, mCRC, Rectal Cancer	Panitumumab, Ganitumab, Rilotumumab	In the clinical trial part 1, no DLT was reported. In part 2, for the panitumumab plus rilotumumab, panitumumab plus ganitumab, and the panitumumab plus placebo arms, the ORRs were 31, 22, and 21%, respectively. Median PFS was 5.2, 5.3, and 3.7 months and median OS 13.8, 10.6, and 11.6 months, respectively. Adverse events were tolerable (5).
NCT00851136; Genentech, Inc.	A Phase Ib Study of the Safety and Pharmacokinetics of PRO95780 Administered in Combination with the FOLFOX Regimen and Bevacizumab in Patients with Previously Untreated, Locally Advanced, Recurrent, and Metastatic Colorectal Cancer; Phase I	Completed; February 2009; June 2010; July 2010	mCRC	Bevacizumab, Drozitumab; FOLFOX	Nine patients were treated at 2 different cohort dose levels of drozitumab. No DLT occurred at either dose level and the maximum tolerated dose was not reached. Two patients had a partial response of 4.93 and 4.96 months duration (6).
NCT00861419; Amgen	An Open-Label, Dose Escalation Study to Evaluate the Safety, Tolerability, and Pharmacokinetics of AMG 386 With AMG 706, AMG 386 With Bevacizumab, AMG 386 With Sorafenib, and AMG 386 With Sunitinib in Adult Patients with Advanced Solid Tumors; Phase I	Completed; March 2009; Not provided; February 2017	Advanced Solid Tumors	Bevacizumab; Sorafenib, Motesanib (AMG 706), Trebananib (AMG 386), Sunitinib	Across the trebananib plus bevacizumab cohorts, 2 patients had a partial response; 11 patients had stable disease lasting >6 months. Across the trebananib plus motesanib cohorts, 1 patient had a partial response; 5 patients had stable disease lasting >6 months. Trebananib plus bevacizumab or motesanib in advanced solid tumors may be associated with less severe toxicities relative to those arising when two anti-VEGF agents are combined (7).
NCT00888043; Herbert Hurwitz, MD	A Phase I/Biomarker Study of Bevacizumab in Combination with CNTO 95 in Patients with Refractory Solid Tumors; Phase I	Completed; April 2009; January 2015; March 2015	Solid Tumors	Intetumubab, Bevacizumab	No tumor responses were noted. Bevacizumab and intetumumab can be administered safely in combination. This treatment resulted in changes in plasma levels of several extracellular matrix-interacting proteins and angiogenic factors (8).
NCT00941928; M.D. Anderson Cancer Center	Adoptive Transfer of Haploidentical NK Cells in Combination with Epratuzumab for the Treatment of Relapsed Acute Lymphoblastic Leukemia; Phase II	Terminated; July 2009; May 2012; May 2014	Leukemia Pediatric Cancer	Epratuzumab; Fludarabine, Cyclophosphamide, Mesna, NK cells, Interleukin-2	Only 2 participants were recruited. Both participants had recurrent disease before single-month assessment and therefore no data were analyzed. Study was terminated early due to slow accrual (Source: www.clinicaltrials.gov).
NCT00954642; Genentech, Inc.	A Phase Ib, Open-Label, Dose-Escalation Study of the Safety and Pharmacology of MNRP1685A, a Human IgG1 Antibody, in Combination with Bevacizumab with or Without Paclitaxel in Patients with Locally Advanced or	Completed; August 2009; December 2011; May 2017	Solid Cancers	Bevacizumab, MNRP1685A; Paclitaxel	Two confirmed and three unconfirmed partial responses were observed. The safety profiles were consistent with the single-agent profiles of all study drugs. However, a higher than expected rate of clinically significant proteinuria was observed, which does not support further

	Metastatic Solid Tumors;<br>Phase I				testing of MNRP1685A in combination with bevacizumab (9)
NCT00989586;<br>Beth Christian	A Phase I/II Study of Veltuzumab (IMMU-106, hA20), a Humanized Anti-CD20 Monoclonal Antibody, Combined with Milatuzumab (IMMU-115, hLL1), a Humanized Anti-CD74 Monoclonal Antibody, in Relapsed and Refractory B-cell Non-Hodgkin's Lymphoma;<br>Phase I, II	Completed;<br>October 2009;<br>September 2015;<br>February 2017	Cancer	Milatuzumab,<br>Veltuzumab	No DLT were observed in the phase I study. Median weeks of therapy was 12 and 29% of patients completed all 36 weeks of therapy. The ORR was 24%, median duration of response was 12 months, and responses were observed at all dose levels and in 50% of patients refractory to rituximab. Combination therapy with veltuzumab and milatuzumab demonstrated activity in a population of heavily pre-treated patients with relapsed or refractory indolent NHL (10).
NCT01008475;<br>Merck KGaA	An Open-label, Randomized, Controlled, Multicenter, Phase I/II Trial Investigating 2 EMD 525797 Doses in Combination with Cetuximab and Irinotecan Versus Cetuximab and Irinotecan Alone, as Second-line Treatment for Subjects With K-ras Wild Type Metastatic Colorectal Cancer (mCRC);<br>Phase I, II	Completed;<br>November 2009;<br>April 2015;<br>March 2016	mCRC	EMD 525797,<br>Cetuximab;<br>Irinotecan	Phase I showed that abituzumab doses up to 1000 mg were well tolerated in combination with SoC. Grade ≥3 treatment-emergent adverse events were observed in 72% of patients. The primary PFS end point was not met, although predefined exploratory biomarker analyses identified subgroups of patients in whom abituzumab may have benefit. The tolerability of abituzumab combined with cetuximab and irinotecan was acceptable (11).
NCT01097460;<br>Merrimack Pharmaceuticals	MM-111-02-12-02: A Phase 1 Study of MM-111 in Combination with Herceptin in Patients With Advanced, Refractory Her2 Amplified, Heregulin Positive Breast Cancer;<br>Phase I	Completed;<br>April 2010;<br>July 2013;<br>January 2015	BC	Trastuzumab, MM-111;<br>Paclitaxel,<br>Carboplatin	A total of 16 participants (female) completed this study (median age 54). Two participants had serious AE. No statistical analysis provided for Incidence of treatment-emergent AEs (Source: www.clinicaltrials.gov).
NCT01248949;<br>MedImmune LLC	Phase 1/1b, Open-Label, Dose-Escalation and Expansion Study to Evaluate the Safety and Antitumor Activity of MEDI3617 as a Single-Agent or in Combination Therapy in Adult Subjects with Advanced Solid Tumors;<br>Phase I	Completed;<br>November 2010;<br>October 2015;<br>March 2017	Advanced Solid Tumors, Advanced Recurrent Ovarian Tumors	MEDI3617,<br>Bevacizumab	MEDI3617 alone and in combination had an acceptable safety profile. Combination therapy exhibited signs of antitumor activity across a variety of tumor types (12).
NCT01307891;<br>University of Alabama at Birmingham	An Open Label, Randomized, Phase II Trial of Abraxane (Paclitaxel Albumin-Bound Particles for Injectable Suspension), With or Without Tigatuzumab (a Humanized Monoclonal Antibody Targeting Death Receptor 5) in Patients with Metastatic, Triple Negative (ER, PR, and HER-2 Negative) Breast	Completed;<br>March 2011;<br>June 2017;<br>October 2017	BC, TNBC, mBC	Tigatuzumab;<br>Abraxane (nab-paclitaxel/ nanoparticle)	Grade 3 toxicities were less than 29%, with no grade 4-5. CTC (circulating tumor cells) were detected in approximately one-third of triple-negative metastatic breast cancer patients. Elevated CTC at baseline and days 15 and 29 were prognostic, and reductions in CTC levels reflected response (13).

	Cancer;<br>Phase II				
NCT01317901;<br>Aptevo Therapeutics	A Phase 1 Study of TRU-016 in Combination with Rituximab and Bendamustine in Subjects with Relapsed Indolent Lymphoma;<br>Phase I	Completed;<br>March 2011;<br>June 2013;<br>June 2017	B-cell Small Lymphocytic Lymphoma	Rituximab, TRU-016; Bendamustine	A cohort of 12 patients started and completed the study, 5 of them showed a complete response. Otlertuzumab in combination with rituximab and bendamustine was well tolerated and induced responses in the majority of patients with relapsed indolent B-NHL (14).
NCT01332721;<br>Tracon Pharmaceuticals Inc.	An Open Label Phase 1B Dose-Escalation Study of TRC105 Combined with Standard-Dose Bevacizumab for Advanced Solid Tumors;<br>Phase I	Completed;<br>April 2011;<br>March 2014;<br>March 2014	Solid Tumors	Bevacizumab, TRC105	Escalating doses of TRC105 were studied in combination with standard-dose bevacizumab in a cohort of 38 patients. The combination was well tolerated and clinical activity was observed in a VEGF inhibitor-refractory population (15).
NCT01399684;<br>Genentech, Inc.	A Phase II, Multicenter, Randomized, Double-Blind, Placebo-Controlled Study Evaluating the Efficacy and Safety of MEGF0444A Dosed to Progression in Combination with Bevacizumab and FOLFOX in Patients with Previously Untreated Metastatic Colorectal Cancer;<br>Phase II	Completed;<br>July 2011;<br>February 2014;<br>August 2016	mCRC	Bevacizumab, Parsatuzumab; 5-Fluorouracil, Folinic acid, Oxaliplatin	The median PFS was 12 months for the experimental arm versus 11.9 months for the control arm. The ORR was 59% in the parsatuzumab arm and 64% in the placebo arm. The adverse event profile was similar in both arms. There was no evidence of efficacy for the addition of parsatuzumab to the combination of bevacizumab and chemotherapy for first-line mCRC (16).
NCT01471210;<br>Bristol-Myers Squibb	A Phase 1 Study of the Safety, Tolerability, Pharmacokinetics and Immunoregulatory Activity of Urelumab (BMS-663513) in Subjects with Advanced and/or Metastatic Solid Tumors and Relapsed/Refractory B-cell Non-Hodgkin's Lymphoma (B-NHL);<br>Phase I	Completed;<br>November 2011;<br>April 2016;<br>April 2017	Solid Tumors, B-Cell Non-Hodgkin's Lymphoma	Urelumab	Urelumab is safe/tolerable, as only 5% of the patients experienced TRAE leading to discontinuation. This antibody stimulated peripheral IFN-γ-induced cytokine production. Six treated patients with lymphoma had a partial (*n* =3) or complete (*n* =3) remission, demonstrating urelumab antitumor activity (17).
NCT01636882;<br>Viralytics	A Phase 2 Study of the Efficacy and Safety of Intratumoral CAVATAK™ (Coxsackievirus A21, CVA21) in Patients with Stage IIIc and Stage IV Malignant Melanoma to Extend Dosing for up to 48 Weeks Total;<br>Phase II	Completed;<br>July 2012;<br>April 2016;<br>August 2017	Melanoma	Coxsackievirus A21, ipilimumab	Combination of CVA21 and ipilimumab was well tolerated and induced anti-tumor activity in local, visceral and non-visceral lesions in a number of patients that had failed previous immunotherapies. Increases in T cell effector and memory subsets were also observed (17).
NCT01688206;<br>Hoffmann-La Roche	An Open-label, Multi-center, Dose Escalation Phase I Study of Single Agent RO5520985 (Vanucizumab), and in Combination with Atezolizumab, Administered as an Intravenous Infusion	Not recruiting participants;<br>September 2012;<br>Estimated:	Neoplasms	Atezolizumab, Vanucizumab	A total of 42 patients were treated on 7 DL: Q2W 3 mg/kg, 6, 12, 19, 30; QW 10 mg/kg, 20, 30. Depletion of circulating targets Ang-2 and VEGF-A was evident and apparently independent of dose and schedule. The progression-free rate at 8 weeks was 67% (Q2W) and

	in Patients with Locally Advanced or Metastatic Solid Tumors; Phase I	November 2017; September 2017			53% (QW). The RP2D and schedule was determined at 30 mg/kg Q2W, where RO5520985 demonstrated an acceptable safety profile with favorable PK and PD effects (18).
NCT01714739; Bristol-Myers Squibb	A Phase 1/2 Study of the Combination of Lirilumab (Anti-KIR) Plus Nivolumab (Anti-PD-1) or Lirilumab Plus Nivolumab and Ipilimumab in Advanced Refractory Solid Tumors; Phase I, II	Recruiting; October 2012; Estimated: February 2021; November 2017	Solid Tumors	Lirilumab, Nivolumab, Ipilimumab	lirilumab plus nivolumab demonstrated a manageable safety profile, as TRAE were reported in 72%, but discontinuations due to TRAE only occurred in 8%. Among evaluable patients, 17% had reductions in tumor burden >80%, with enduring responses. These data demonstrate clinical benefit and a preliminary efficacy of this combination (19).
NCT01727089; National Cancer Institute	A Phase II Study of Bevacizumab Alone or in Combination with TRC105 for Advanced Renal Cell Cancer; Phase II	Completed; November 2012; August 2017; September 2014	Renal Cell Carcinoma, Papillary Renal Cell Carcinoma	Bevacizumab, TRC105	Fifty-nine patients were enrolled. Grade ≥3 toxicities occurred in 16 patients who received bevacizumab compared with 19 who received bevacizumab plus TRC105.TRC105 failed to improve PFS when added to bevacizumab (20).
NCT01775631; Bristol-Myers Squibb	A Phase 1b, Open-label, Multicenter Study of Urelumab (BMS-663513) in Combination with Rituximab in Subjects with Relapsed/Refractory B-cell Malignancies; Phase I	Completed; January 2013; August 2016; March 2017	B-Cell Malignancies	Urelumab, Rituximab	Urelumab is safe and well tolerated in combination with rituximab or cetuximab at doses of 0.1 mg/kg or 8 mg, with minimal evidence of liver toxicity. Although pharmacodynamic activity was observed in peripheral blood samples, urelumab with rituximab or cetuximab did not demonstrate substantial enhancement of clinical responses or lead to intratumoral immune modulation (21).
NCT01968109; Bristol-Myers Squibb	A Phase I/2a Dose Escalation and Cohort Expansion Study of the Safety, Tolerability, and Efficacy of Anti-LAG-3 Monoclonal Antibody (BMS-986016) Administered Alone and in Combination with Anti-PD-1 Monoclonal Antibody (Nivolumab, BMS-936558) in Advanced Solid Tumors; Phase I, II	Recruiting; October 2013; Estimated: October 2019; November 2017	Neoplasms	BMS-986016, Nivolumab	BMS-986016 monotherapy was well tolerated at the dose levels tested. BMS-986016 in combination with nivolumab demonstrated biological activity as evidenced by toxicities characteristic of immune checkpoint blockers and objective tumor regressions (21).
NCT02009449; ARMO BioSciences	A Phase 1, Open-Label Dose Escalation First-in-Human Study to Evaluate the Tolerability, Safety, Maximum Tolerated Dose, Preliminary Clinical Activity and Pharmacokinetics of AM0010 in Patients with Advanced Solid Tumors; Phase I	Active, not recruiting; December 2013; Estimated: March 2018; March 2017	Melanoma, Prostate Cancer, Ovarian Cancer, Renal Cell Carcinoma, Colorectal Carcinoma, Pancreatic Carcinoma, NSCLC, Solid Tumors, BC	Pembrolizumab, Nivolumab; AM0010, Paclitaxel, Docetaxel, Carboplatin, Cisplatin, FOLFOX, Gemcitabine,	AM0010 alone or in combination with anti-PD-1 was well tolerated (all TrAE were transient). AM0010 alone and in combination with anti-PD-1 increased Th1 cytokines, $CD8^+$ T cell-associated effector molecules as well as cytokines stimulating T cell proliferation (21).

				Capecitabine, Pazopanib	
NCT02043665; Merck Sharp & Dohme Corp. / Viralytics	A Phase 1, Dose-finding and Signal-seeking Study of the Safety and Efficacy of Intravenous CAVATAK® (Coxsackievirus A21, CVA21) Alone and in Combination with Pembrolizumab in Patients With Late Stage Solid Tumours (NSCLC, Castrate-resistant Prostate Cancer, Melanoma, Bladder Cancer); Phase I	Recruiting; January 2014; Estimated: August 2019; September 2017	NSCLC, Bladder Cancer, Melanoma, Prostate Cancer	Pembrolizumab; Coxsackievirus A21	Delivery of CVA21 and pembrolizumab to all patients was generally well tolerated, with no Grade 3 or 4 product-related AE. CVA21 tumor targeting in patients with melanoma, NSCLC, and bladder cancer patients was confirmed by detection of CVA21 viral RNA in tumor biopsies at study day 8 (21).
NCT02061761; Bristol-Myers Squibb	A Phase 1/2a Dose Escalation and Cohort Expansion Study of the Safety, Tolerability, and Efficacy of Anti-LAG-3 (BMS-986016) in Monoclonal Antibody (BMS-986016) Administered Alone and in Combination with Anti-PD-1 Monoclonal Antibody (Nivolumab, BMS-936558) in Relapsed or Refractory B-Cell Malignancies; Phase I, II	Recruiting; February 2014; Estimated: January 2020; November 2017	Hematologic Neoplasms	BMS-986016, Nivolumab	BMS-986016 monotherapy was well tolerated at the dose levels tested. BMS-986016 in combination with nivolumab demonstrated biological activity as evidenced by toxicities characteristic of immune checkpoint blockers and objective tumor regressions (21).
NCT02110082; Bristol-Myers Squibb	A Phase 1b, Open-label, Multicenter Study of Urelumab (BMS-663513) in Combination with Cetuximab in Subjects with Advanced/Metastatic Colorectal Cancer or Advanced/Metastatic Squamous Cell Carcinoma of the Head and Neck; Phase I	Completed; April 2014; December 2016; April 2017	CRC, Head and Neck Cancer	Urelumab, Cetuximab	Urelumab is safe and well tolerated in combination with rituximab or cetuximab at doses of 0.1 mg/kg or 8 mg, with minimal evidence of liver toxicity. Although pharmacodynamics activity was observed in peripheral blood samples, urelumab with rituximab or cetuximab did not demonstrate substantial enhancement of clinical responses or lead to intratumoral immune modulation (21).
NCT02132403; University of Illinois at Chicago / Biothera	PM-01: Phase 1b Study of PGG Beta Glucan (Imprime PGG) in Combination With Anti-MUC1 Antibody (BTH1704) and Gemcitabine (Gemzar) for the Treatment of Advanced Pancreatic Cancer; Phase I	Terminated (Drug recall); May 2014; April 2015; May 2015	Pancreatic Cancer	BTH1704; IMPRIME PGG, Gemcitabine	A phase I/II trial of Imprime PGG in combination with an antibody and gemcitabine in pancreatic cancer was terminated early due to a drug recall (drug not specified) (22).
NCT02179918; Pfizer / Merck Sharp & Dohme Corp.	A Phase 1b Study of the 4-1bb Agonist Pf-05082566 In Combination with The Pd-1 Inhibitor Mk-3475 In Patients with Advanced Solid Tumors; Phase I	Completed; July 2014; February 2017; March 2017	Advanced Solid Tumors	PF-05082566, MK-3475	Twenty-three patients received combination treatment with no dose-limiting toxicities. TrAE were mostly grades 1 to 2. Six patients had confirmed complete or partial responses. A trend toward higher levels of activated memory/effector peripheral blood $CD8^+$ T cells was observed in responders versus non-responders.

					Utomilumab in combination with pembrolizumab demonstrated safety, tolerability and clinical activity (23).
NCT02253992; Bristol-Myers Squibb	A Phase 1/2 Dose Escalation and Cohort Expansion Study of the Safety and Tolerability of Urelumab Administered in Combination with Nivolumab in Advanced/Metastatic Solid Tumors and B-cell Non-Hodgkins Lymphoma; Phase I, II	Recruiting; October 2014; Estimated: September 2019; November 2017	Advanced Solid Tumors, Advanced B-cell NHL	Urelumab, Nivolumab	Urelumab alone or with nivolumab was safe/tolerable at flat and weight-based doses of 8 mg and 0.1 mg/kg. Although urelumab demonstrated single-agent pharmacodynamics and antitumor activity in lymphoma, combination with nivolumab did not appear to provide significant additive/synergistic clinical benefit at the doses evaluated (17).
NCT02272855; Takara Bio Inc. /Theradex	A Phase II Study of Combination Treatment with HF10, a Replication-competent HSV-1 Oncolytic Virus, and Ipilimumab in Patients With Stage IIIB, Stage IIIC, or Stage IV Unresectable or Metastatic Malignant Melanoma; Phase II	Active, not recruiting; October 2014; Estimated: December 2017; October 2017	Malignant Melanoma	Ipilimumab; HF10	Combination of Ipilimumab and HF10 showed a beneficial therapeutic effect as second-line therapy. The best overall response was 41% and clinical therapeutic efficacy was 68%. Median progression-free survival was 19 months and median overall survival was 21.8 months (24).
NCT02410512; Genentech, Inc.	A Phase Ib, Open-Label, Dose-Escalation Study of the Safety and Pharmacokinetics of MOXR0916 and Atezolizumab in Patients with Locally Advanced or Metastatic Solid Tumors; Phase I	Recruiting; April 2015; Estimated: August 2018; November 2017	Neoplasms	Atezolizumab, MOXR0916	A combination regimen of the PD-L1 inhibitor atezolizumab (Tecentriq) and the investigational OX40 agonist MOXR0916 was well tolerated and showed early signs of antitumor activity in solid tumors, according to results from a Phase Ib dose escalation study (25).
NCT02448810; Baxalta now part of Shire	A Phase 2a Randomized, Open-label Study to Assess the Safety, Tolerability, and Efficacy of BAX69 in Combination With 5-FU/Leucovorin or Panitumumab Versus Standard of Care in Subjects with Metastatic Colorectal Cancer; Phase II	Terminated; May 2015; February 2017; October 2017	Metastatic Colorectal Cancer	BAX69, Panitumumab; 5-FU/LV	In the Phase IIa study, there were no DLT or related SAE reported among the 13 patients treated. Based on the Phase I and Phase II studies, imalumab administered intravenously weekly in a 28-day cycle alone or in combination with either 5-FU/LV or panitumumab is generally safe and well-tolerated (26).
NCT02493361; Alain Algazi	An Open-label, Phase II, Multicenter Study of Enhancing Pembrolizumab Responses in Melanoma Through Intratumoral pIL-12 Electroporation; Phase II	Active, not recruiting; July 2015; Estimated: March 2019; November 2017	Melanoma	Pembrolizumab; pIL-12	The combination IT-pIL12-EP with pembrolizumab in patients with an anti-PD-1 non-responsive phenotype engendered a 40% clinical response with associated positive immune-based biomarker data and an excellent safety profile. This suggests that IT-pIL12-EP modulates the tumor microenvironment to enable an effective anti-PD-1 mAb response in patients otherwise unlikely to respond (19).
NCT02495636; Yale University	Phase 2 Study of MPDL3280A Combined With CDX-1401 in NY-ESO 1 (+) IIIB, IV or Recurrent Non-Small Cell	Withdrawn prior to enrollment (Study closed	NSCLC	Atezolizumab (MPDL3280A); poly-ICLC	Clinical trial withdrawn prior to enrollment (Source: www.clinicaltrials.gov).

	Lung Cancer;<br>Phase II	due to lack of enrollment);<br>July 2015;<br>Not provided;<br>June 2017		(Personalized Genomic Vaccine 001), DEC-205/NY-ESO-1 Fusion Protein CDX-1401	
NCT03050814;<br>NCI	A Randomized Phase II Trial of Standard of Care Alone or in Combination With Ad-CEA Vaccine and Avelumab in Patients With Previously Untreated Metastatic Colorectal Cancer;<br>Phase II	This study has suspended participant recruitment;<br>February 2017;<br>Estimated:<br>August 2021;<br>August 2017	CRC	Avelumab, Bevacizumab;<br>Ad-CEA vaccine, 5-FU, Leucovorin, Oxaliplatin, Capecitabine	This study has a stopping rule when death occurs within 30 days of receiving investigational drug; study has been closed until further discussion (Source: www.clinicaltrials.gov).

1) *Abbreviations*: AE, adverse events; BC, breast cancer; CRC, colorectal cancer; CI, confidence interval; CLC, cell lung cancer; DL, drug loading; DLT, dose limiting toxicity; DTH, delayed type hypersensitivity; irAE, immune-related adverse events; mBC, metastatic breast cancer; mCRC, metastatic colorectal cancer; MM, multiple myeloma; NCI, National Cancer Institute; NSCLC, non-small cell lung cancer; ORR, overall response rate; OS, overall survival; PD, pharmacodynamics; PK, pharmacokinetics; pts, patients; PFS, progression-free survival; PSA, prostate-specific antigen; QW, once-weekly; RFS, recurrence-free survival; RP2D, recommended phase II dose; SAE, serious adverse event; SoC, standard of care; SCS, squamous cell carcinoma; TNBC, triple negative breast cancer; TrAE, treatment-related adverse event.

References Cited

1. Posner MC, Niedzwiecki D, Venook AP, Hollis DR, Kindler HL, Martin EW, et al. A phase II prospective multi-institutional trial of adjuvant active specific immunotherapy following curative resection of colorectal cancer hepatic metastases: cancer and leukemia group B study 89903. *Ann Surg Oncol* (2008) 15(1):158-64. doi: 10.1245/s10434-007-9654-7. PubMed PMID: 18008108.

2. Madan RA, Mohebtash M, Arlen PM, Vergati M, Rauckhorst M, Steinberg SM, et al. Ipilimumab and a poxviral vaccine targeting prostate-specific antigen in metastatic castration-resistant prostate cancer: a phase 1 dose-escalation trial. *Lancet Oncol* (2012) 13(5):501-8. doi: 10.1016/S1470-2045(12)70006-2. PubMed PMID: 22326924.

3. Kim ES, Moon J, Herbst RS, Redman MW, Dakhil SR, Velasco MR, Jr., et al. Phase II trial of carboplatin, paclitaxel, cetuximab, and bevacizumab followed by cetuximab and bevacizumab in advanced nonsquamous non-small-cell lung cancer: SWOG S0536. *J Thorac Oncol* (2013) 8(12):1519-28. doi: 10.1097/JTO.0000000000000009. PubMed PMID: 24189513; PubMed Central PMCID: PMCPMC4072123.

4. Chen G, Gupta R, Petrik S, Laiko M, Leatherman JM, Asquith JM, et al. A feasibility study of cyclophosphamide, trastuzumab, and an allogeneic GM-CSF-secreting breast tumor vaccine for HER2+ metastatic breast cancer. *Cancer Immunol Res* (2014) 2(10):949-61. doi: 10.1158/2326-6066.CIR-14-0058. PubMed PMID: 25116755; PubMed Central PMCID: PMCPMC4211036.

5. Van Cutsem E, Eng C, Nowara E, Swieboda-Sadlej A, Tebbutt NC, Mitchell E, et al. Randomized phase Ib/II trial of rilotumumab or ganitumab with panitumumab versus panitumumab alone in patients with wild-type KRAS metastatic colorectal cancer. *Clin Cancer Res* (2014) 20(16):4240-50. doi: 10.1158/1078-0432.CCR-13-2752. PubMed PMID: 24919569; PubMed Central PMCID: PMCPMC4371780.

6. Rocha Lima CM, Bayraktar S, Flores AM, MacIntyre J, Montero A, Baranda JC, et al. Phase Ib study of drozitumab combined with first-line mFOLFOX6 plus bevacizumab in patients with metastatic colorectal cancer. *Cancer Invest* (2012) 30(10):727-31. doi: 10.3109/07357907.2012.732163. PubMed PMID: 23061802.

7. Hong DS, Gordon MS, Samlowski WE, Kurzrock R, Tannir N, Friedland D, et al. A phase I, open-label study of trebananib combined with sorafenib or sunitinib in patients with advanced renal cell carcinoma. *Clin Genitourin Cancer* (2014) 12(3):167-77 e2. doi: 10.1016/j.clgc.2013.11.007. PubMed PMID: 24365125; PubMed Central PMCID: PMCPMC4754667.

8. Uronis HE, Jia J, Bendell JC, Howard L, Ready NA, Lee PH, et al. A Phase I/biomarker study of bevacizumab in combination with CNTO 95 in patients with advanced solid tumors. *Cancer Chemother Pharmacol* (2015) 75(2):343-52. doi: 10.1007/s00280-014-2647-x. PubMed PMID: 25527204.

9. Patnaik A, LoRusso PM, Messersmith WA, Papadopoulos KP, Gore L, Beeram M, et al. A Phase Ib study evaluating MNRP1685A, a fully human anti-NRP1 monoclonal antibody, in combination with bevacizumab and paclitaxel in patients with advanced solid tumors. *Cancer Chemother Pharmacol* (2014) 73(5):951-60. doi: 10.1007/s00280-014-2426-8. PubMed PMID: 24633809.

10. Christian BA, Poi M, Jones JA, Porcu P, Maddocks K, Flynn JM, et al. The combination of milatuzumab, a humanized anti-CD74 antibody, and veltuzumab, a humanized anti-CD20 antibody, demonstrates activity in patients with relapsed and refractory B-cell non-Hodgkin lymphoma. *Br J Haematol* (2015) 169(5):701-10. doi: 10.1111/bjh.13354. PubMed PMID: 25847298.

11. Elez E, Kocakova I, Hohler T, Martens UM, Bokemeyer C, Van Cutsem E, et al. Abituzumab combined with cetuximab plus irinotecan versus cetuximab plus irinotecan alone for patients with KRAS wild-type metastatic colorectal cancer: the randomised phase I/II POSEIDON trial. *Ann Oncol* (2015) 26(1):132-40. doi: 10.1093/annonc/mdu474. PubMed PMID: 25319061.

12. Hyman DM RN, Natale RB, Armstrong DK, Birrer MJ, Recht LD, et al. A phase 1 study of MEDI3617, a selective angiopoietin-2 inhibitor, alone and in combination with carboplatin/paclitaxel, paclitaxel, or bevacizumab in patients with advanced solid tumors [abstract]. . *J Clin Oncol* (2014) 32(15 suppl):3012.

13. Paoletti C, Li Y, Muniz MC, Kidwell KM, Aung K, Thomas DG, et al. Significance of Circulating Tumor Cells in Metastatic Triple-Negative Breast Cancer Patients within a Randomized, Phase II Trial: TBCRC 019. *Clin Cancer Res* (2015) 21(12):2771-9. doi: 10.1158/1078-0432.CCR-14-2781. PubMed PMID: 25779948; PubMed Central PMCID: PMCPMC5521206.

14. Gopal AK, Tarantolo SR, Bellam N, Green DJ, Griffin M, Feldman T, et al. Phase 1b study of otlertuzumab (TRU-016), an anti-CD37 monospecific ADAPTIR therapeutic protein, in combination with rituximab and bendamustine in relapsed indolent lymphoma patients. *Invest New Drugs* (2014) 32(6):1213-25. doi: 10.1007/s10637-014-0125-2. PubMed PMID: 24927856; PubMed Central PMCID: PMCPMC4229440.

15. Gordon MS, Robert F, Matei D, Mendelson DS, Goldman JW, Chiorean EG, et al. An open-label phase Ib dose-escalation study of TRC105 (anti-endoglin antibody) with bevacizumab in patients with advanced cancer. *Clin Cancer Res* (2014) 20(23):5918-26. doi: 10.1158/1078-0432.CCR-14-1143. PubMed PMID: 25261556; PubMed Central PMCID: PMCPMC4570619.

16. Garcia-Carbonero R, van Cutsem E, Rivera F, Jassem J, Gore I, Jr., Tebbutt N, et al. Randomized Phase II Trial of Parsatuzumab (Anti-EGFL7) or Placebo in Combination with FOLFOX and Bevacizumab for First-Line Metastatic Colorectal Cancer. *Oncologist* (2017) 22(4):375-e30. doi: 10.1634/theoncologist.2016-0133. PubMed PMID: 28275117; PubMed Central PMCID: PMCPMC5388369.

17. Lundqvist A, van Hoef V, Zhang X, Wennerberg E, Lorent J, Witt K, et al. 31st Annual Meeting and Associated Programs of the Society for Immunotherapy of Cancer (SITC 2016): part one. *Journal for ImmunoTherapy of Cancer* (2016) 4(1):82. doi: 10.1186/s40425-016-0172-7.

18. Hidalgo M, Tourneau CL, Massard C, Boni V, Calvo E, Albanell J, et al. Results from the first-in-human (FIH) phase I study of RO5520985 (RG7221), a novel bispecific human anti-ANG-2/anti-VEGF-A antibody, administered as an intravenous infusion to patients with advanced solid tumors. *Journal of Clinical Oncology* (2014) 32(15_suppl):2525-. doi: 10.1200/jco.2014.32.15_suppl.2525.

19. Althammer S, Steele K, Rebelatto M, Tan TH, Wiestler T, Schmidt G, et al. 31st Annual Meeting and Associated Programs of the Society for Immunotherapy of Cancer (SITC 2016): late breaking abstracts: National Harbor, MD, USA. 9-13 November 2016. *Journal for Immunotherapy of Cancer* (2016) 4(Suppl 2):91. doi: 10.1186/s40425-016-0191-4. PubMed PMID: PMC5260784.

20. Dorff TB, Longmate JA, Pal SK, Stadler WM, Fishman MN, Vaishampayan UN, et al. Bevacizumab alone or in combination with TRC105 for patients with refractory metastatic renal cell cancer. *Cancer* (2017). doi: 10.1002/cncr.30942. PubMed PMID: 28832978.

21. Ager C, Reilley M, Nicholas C, Bartkowiak T, Jaiswal A, Curran M, et al. 31st Annual Meeting and Associated Programs of the Society for Immunotherapy of Cancer (SITC 2016): part two. *Journal for ImmunoTherapy of Cancer* (2016) 4(1):73. doi: 10.1186/s40425-016-0173-6.

22. Barton C, Vigor K, Scott R, Jones P, Lentfer H, Bax HJ, et al. Beta-glucan contamination of pharmaceutical products: How much should we accept? *Cancer Immunol Immunother* (2016) 65(11):1289-301. doi: 10.1007/s00262-016-1875-9. PubMed PMID: 27473075; PubMed Central PMCID: PMCPMC5069311.

23. Tolcher AW, Sznol M, Hu-Lieskovan S, Papadopoulos KP, Patnaik A, Rasco DW, et al. Phase Ib Study of Utomilumab (PF-05082566), a 4-1BB/CD137 Agonist, in Combination with Pembrolizumab (MK-3475) in Patients with Advanced Solid Tumors. *Clin Cancer Res* (2017). doi: 10.1158/1078-0432.CCR-17-1243. PubMed PMID: 28634283.

24. Eissa IR, Naoe Y, Bustos-Villalobos I, Ichinose T, Tanaka M, Zhiwen W, et al. Genomic Signature of the Natural Oncolytic Herpes Simplex Virus HF10 and Its Therapeutic Role in Preclinical and Clinical Trials. *Front Oncol* (2017) 7:149. doi: 10.3389/fonc.2017.00149. PubMed PMID: 28770166; PubMed Central PMCID: PMCPMC5509757.

25. Infante JR, et al. A Phase lb Dose Escalation Study of the OX40 Agonist MOXR0916 and the PD-L1 Inhibitor Atezolizumab in Patients with Advanced Solid Tumors. *J Clin Oncol* (2016) (34, (suppl; abstr 101)).

26. Mahalingam D, et al. Safety and efficacy analysis of imalumab, an anti-oxidized macrophage migration inhibitory factor (oxMIF) antibody, alone or in combination with 5-fluorouracil/leucovorin (5-FU/LV) or panitumumab, in patients with metastatic colorectal cancer (mCRC) *Annals of Oncology* (2016) 27(Issue suppl_2, 1 June 2016):ii105. doi: https://doi.org/10.1093/annonc/mdw200.11.
