# Supplementary material for: New Strategies Using Antibody Combinations to Increase Cancer Treatment Effectiveness
Source: Front Immunol. 2017 Dec 21;8:1804. doi: 10.3389/fimmu.2017.01804 (PMC5742572; doi:10.3389/fimmu.2017.01804)
Supplement: Supplementary file 2 [file table_2.PDF]

Suppl. Table II: Summarized characteristics of selected clinical trials including antibodies in combination with other biologicals for cancer therapy without results posted on ClinicalTrial.gov

Clinical trial identifier; Sponsor and collaborators	Official Title; Phase	Study status; First posted; Completion date; Last Update	Condition	Antibody; Other drugs, biologics
NCT00003082; University of Arizona, NCI[1]	A Phase IA Trial of Combined Murine IgG Monoclonal Anti-Transferrin Receptor Antibodies E2.3 and A27.15 in Cancer Patients; Phase I	Completed; July 2004; February 2001; March 2010	Chronic Myeloproliferative Disorders, Leukemia, Lymphoma Multiple, Myeloma and Plasma Cell Neoplasm, Myelodysplastic Syndromes, Precancerous/Nonmalignant Condition Unspecified Adult Solid Tumor, Protocol Specific	A27.15, E2.3
NCT00009750; University of California / Davis	Combined Modality Radioimmunotherapy For Hormone Refractory Metastatic Prostate Cancer with Two Cycles of Escalating Dose 90Y-DOTA-Peptide-m170 And Fixed, Low Dose Paclitaxel with Blood Stem Cell Support and Cyclosporin For HAMA Suppression; Phase I	Unknown; January 2003; Not Provided; September 2013	Prostate Cancer	m170, Indium In 111 mAb m170, Yttrium Y 90 mAb m170; Filgrastim, Cyclosporine, Paclitaxel, Peripheral Blood Stem Cell Transplantation
NCT00028535; NCI	Phase I Trial of Interleukin-12 in Combination with Paclitaxel Plus Herceptin in Patients with Her2-positive Malignancies; Phase I	Completed 2003 Primary; February 2009; June 2013	Male BC, Recurrent BC, Recurrent Endometrial Carcinoma, Recurrent Gastric Cancer, Recurrent NSCLC, Recurrent OC, Epithelial Cancer, Recurrent SCLC	Trastuzumab; Paclitaxel, Recombinant Interleukin-12
NCT00045617; Southwest Oncology Group / NCI	A Phase II Trial of Patients with Limited Stage SCLC Treated with Thoracic Radiation Therapy and Chemotherapy with Cisplatin/Etoposide Followed by Cisplatin/Etoposide and Anti-Idiotype Monoclonal Antibody Vaccines; Phase II	Terminated; January 2003; May 2003; June 2012	Lung Cancer	11D10 Anti-Idiotype Vaccine, GD2 Anti-Idiotype Vaccine; Cisplatin Etoposide Radiation Therapy
NCT00058435; Memorial Sloan Kettering Cancer Center / NCI	Phase I Trial of the Monoclonal Anti-Idiotype Antibody ACA125 in Patients with Epithelial Ovarian, Fallopian Tube, or Peritoneal Cancer; Phase I	Completed; April 2003; March 2004; June 2013	FTC, OC, Primary Peritoneal Cavity Cancer	Abagovomab
NCT00626483; John Sampson	REGULATory T-Cell Inhibition with Basiliximab (Simulect®) During Recovery From Therapeutic Temozolomide-induced Lymphopenia During Antitumor Immunotherapy Targeted Against Cytomegalovirus in Patients with Newly-Diagnosed Glioblastoma	Active, not recruiting participants; February 2008; Estimated: June	Malignant Neoplasms, Brain	Basiliximab; RNA-Loaded Dendritic Cell Vaccine

	Multiforme;<br>Phase I	2018;<br>June 2017		
NCT01051934;<br>NCI	A Phase I Trial of SS1 (dsFv) PE38 with Paclitaxel, Carboplatin, and Bevacizumab in Subjects with Unresectable Non-Small Cell Lung Adenocarcinoma;<br>Phase I	Completed;<br>January 2010;<br>September 2011;<br>July 2017	NSCLC, Adenocarcinoma	SS1 (dsFv) PE38, Bevacizumab;<br>Paclitaxel, Carboplatin, immunotoxin
NCT01067287;<br>Beth Israel Deaconess Medical Center	Blockade of PD-1 in Conjunction with the Dendritic Cell/Myeloma Vaccines Following Stem Cell Transplantation;<br>Phase II	Active, not recruiting participants;<br>February 2010;<br>Estimated Primary: December 2018;<br>July 2017	MM	Pidilizumab;<br>Dendritic Cell Fusion Vaccine
NCT01327612;<br>Amgen	A Phase 2 Open Label Extension Study of Conatumumab and AMG 479;<br>Phase II	Active, not recruiting participants;<br>April 2011;<br>Estimated: January 2018;<br>July 2017	Advanced Solid Tumors, Carcinoid, CRC, Locally Advanced, Lymphoma, Metastatic Cancer, NSCLC, Sarcoma Solid Tumors	Conatumumab, Ganitumab, Bevacizumab;<br>FOLFOX6
NCT01514123;<br>Circadian Technologies Ltd.	A Phase I, Open Label, Dose Escalation Study of the VEGF-C Human Monoclonal Antibody VGX-100 Administered by Intravenous Infusion Alone and Co-administered with Bevacizumab in Adult Subjects with Advanced or Metastatic Solid Tumors;<br>Phase I	Active, not recruiting participants;<br>January 2012;<br>Estimated: November 2017;<br>July 2017	Neoplasms	VGX-100, Bevacizumab
NCT01922921;<br>University of Washington	Phase I/II Randomized Study of Combination Immunotherapy with or Without Polysaccharide Krestin (PSK®) Concurrently with a HER2 ICD Peptide-Based Vaccine in Patients with Stage IV BC Receiving HER2-Targeted Monoclonal Antibody Therapy;<br>Phase I, II	Active, not recruiting participants;<br>August 2013;<br>Estimated Primary;<br>March 2018;<br>October 2017	HER2/Neu Positive, Recurrent BC	Pertuzumab, Trastuzumab;<br>HER-2/neu Intracellular Domain Protein Polysaccharide-K
NCT01969643;<br>Seattle Genetics, Inc.	A Phase 1, Open-Label, Dose-Escalation Study to Evaluate the Safety and Tolerability of SGN-LIV1A in Patients with Metastatic BC;<br>Phase I	Recruiting participants;<br>October 2013;<br>Estimated:<br>December 2019;<br>November 2017	BC	SGN-LIV1A (SLC39A6), Trastuzumab, Blinatumomab
NCT02003222;<br>NCI	A Phase III Randomized Trial of Blinatumomab for Newly Diagnosed BCR-ABL-Negative B Lineage ALL in Adults;<br>Phase III	Recruiting participants;<br>December 2013;	B-ALL, Philadelphia Chromosome Negative BCR/ABL1 Fusion Protein Negative Untreated Adult ALL	Rituximab, Blinatumomab;<br>Allogeneic Hematopoietic Stem Cell Transplantation,

		Estimated Primary: June 2018; October 2017		Cyclophosphamide, Cytarabine, Daunorubicin Hydrochloride, Dexamethasone, Etoposide, Mercaptopurine, Methotrexate, Peg aspargase, Prednisone, Vincristine Sulfate
NCT02076633; Philogen S.p.A.	A Phase II Study of Intratumoral Application of L19IL2/L19TNF in Melanoma Patients in Clinical Stage III or Stage IV M1a with Presence of Injectable Cutaneous and/or Subcutaneous Lesions; Phase II	Completed; March 2014; May 2015; May 2015	Malignant Melanoma, Skin	L19IL2, L19TNF
NCT02082210; Eli Lilly and Company	A Phase 1b/2 Study of Ramucirumab in Combination with LY2875358 in Patients with Advanced Cancer; Phase I, II	Active, not recruiting participants; March 2014; Estimated; December 2017; July 2017	Advanced Cancer, Gastric Adenocarcinoma, Gastroesophageal Junction Adenocarcinoma, Hepatocellular Cancer, RCC, NSCLC	Emibetuzumab, Ramucirumab
NCT02129075; NCI	A Phase II, Open-Label, Multicenter, Randomized Study of CDX-1401, a Dendritic Cell Targeting NY-ESO-1 Vaccine, in Patients with Malignant Melanoma Pre-treated with Recombinant CDX-301, a Recombinant Human Flt3 Ligand; Phase II	Active, not recruiting participants; May 2014; Primary: April 2016; November 2016	Melanoma	DEC-205/NY-ESO-1 Fusion Protein, CDX-1401; Neoantigen-Based Melanoma-Poly-ICLC Vaccine, Recombinant Flt3 Ligand
NCT02141542; F. Stephen Hodi, MD	Phase I Clinical Trial of Tremelimumab Plus MEDI3617 in Patients with Unresectable Stage III or Stage IV Melanoma; Phase I	Active, not recruiting participants; May 2014; Estimated: February 2019; July 2017	Metastatic Melanoma	Tremelimumab; MEDI3617
NCT02173093; Barbara Ann Karmanos Cancer Institute	Treatment of Neuroblastoma and GD2-Positive Tumors with Activated T Cells Armed with OKT3 X Humanized 3F8 Bispecific Antibodies (GD2Bi): A Phase I/II Study; Phase I, II	Recruiting participants; June 2014; Estimated: May 2018; August 2017	Desmoplastic Small Round Cell Tumor, Disseminated Neuroblastoma, Metastatic Osteosarcoma, Recurrent Neuroblastoma, Recurrent Osteosarcoma	GD2Bi-aATC; IL-2, GM-CSF

NCT02174172; Hoffmann-La Roche	A Phase Ib Study of The Safety and Pharmacology of Atezolizumab (Anti–PD-L1 Antibody) Administered with Ipilimumab, Interferon-Alpha, or Other Immune-Modulating Therapies in Patients with Locally Advanced or Metastatic Solid Tumors; Phase I	Recruiting participants; June 2014; Estimated: February 2019; August 2017	Solid Cancers	Atezolizumab, Bevacizumab, Ipilimumab, Obinutuzumab; Interferon alfa-2b, PEG-interferon alfa-2a
NCT02270372; Cascadian Therapeutics Inc.	A Phase 1b Study of ONT 10 and Varlilumab in Patients with Advanced Ovarian Cancer or BC; Phase I	Completed; October 2014; June 2016; August 2016	Advanced BC, Advanced OC	Varlilumab; ONT-10
NCT02277197; James J Lee	A Phase 1b Study of Ficlatuzumab and Cetuximab in Recurrent/Metastatic HNSCC with Biomarker Correlatives; Phase I	Active, not recruiting participants; October 2014; Estimated: June 2020; September 2016	Squamous Cell Carcinoma of the HNC	Ficlatuzumab, Cetuximab
NCT02291055; Advaxis, Inc.	Phase 1-2 Study of ADXS11-001 or MEDI4736 Alone or Combination in Previously Treated Locally Advanced or Metastatic Cervical or HPV+ Head & Neck Cancer; Phase I, II	Recruiting participants; November 2014; Estimated: December 2019; January 2017	Cervical Cancer, HNC	Durvalumab; ADXS11-001
NCT02302339; Celldex Therapeutics	A Phase 2 Study of Glembatumumab Vedotin, an Anti-gpNMB Antibody-drug Conjugate, as Monotherapy or in Combination with Immunotherapies in Patients with Advanced Melanoma; Phase II	Recruiting participants; November 2014; Estimated: June 2020; June 2017	Melanoma	Glembatumumab vedotin, Nivolumab, Pembrolizumab
NCT02341625; Bristol-Myers Squibb	A Phase I/IIa Study of BMS-986148, a Mesothelin Directed Antibody Drug Conjugate, in Subjects with Select Advanced Solid Tumors; Phase I, II	Active, not recruiting participants; January 2015; Estimated: August 2022; October 2017	Advanced Solid Tumors	BMS-986148, Nivolumab
NCT02350673; Hoffmann-La Roche	A Phase 1b, Open-Label, Multi-Center, Dose Escalation Study of the Safety, Pharmacokinetics, and Therapeutic Activity of Cergutuzumab Amunaleukin, an Immunocytokine, Which Consists of a Variant of Interleukin 2 (IL 2v), That Targets Carcinoembryonic Antigen (CEA), and Atezolizumab, an Antibody That	Recruiting participants; January 2015; Estimated:	Solid Tumors	Atezolizumab, Cergutuzumab amunaleukin, Obinutuzumab

	Targets Programmed Death-Ligand 1 (PD-L1), Administered Intravenously, in Patients with Locally Advanced and/or Metastatic Solid Tumors;<br>Phase I	December 2018;<br>October 2017		
NCT02367196;<br>Celgene	A Phase I, Open-Label, Dose Finding Study of CC-90002, a Monoclonal Antibody Directed Against CD47, in Subjects with Advanced Solid and Hematologic Cancers;<br>Phase I	Recruiting participants;<br>February 2015;<br>Estimated: June 2019;<br>October 2017	Hematologic Neoplasms	CC-90002, Rituximab
NCT02410512;<br>Genentech, Inc.	A Phase Ib, Open-Label, Dose-Escalation Study of the Safety and Pharmacokinetics of MOXR0916 and Atezolizumab in Patients with Locally Advanced or Metastatic Solid Tumors;<br>Phase I	Recruiting participants;<br>April 2015;<br>Estimated: August 2018;<br>October 2017	Neoplasms	Atezolizumab, MOXR0916
NCT02448810;<br>Baxalta US Inc.	A Phase 2a Randomized, Open-label Study to Assess the Safety, Tolerability, and Efficacy of BAX69 in Combination with 5-FU/Leucovorin or Panitumumab Versus Standard of Care in Subjects with Metastatic CRC;<br>Phase II	Terminated (Based on overall benefit-risk assessment);<br>May 2015;<br>February 2017;<br>October 2017	Metastatic CRC	Imalumab (BAX69),<br>Panitumumab;<br>5-FU/LV
NCT02451930;<br>Eli Lilly and Company	An Open-Label, Multicenter, Phase 1b Study with an Expansion Cohort to Evaluate Safety and Efficacy of the Combination of Necitumumab with Pembrolizumab in Patients with Stage IV NSCLC;<br>Phase I	Active, not recruiting participants;<br>May 2015;<br>Estimated: December 2017;<br>February 2017	NSCLC	Necitumumab,<br>Pembrolizumab
NCT02466568;<br>H. Lee Moffitt Cancer Center and Research Institute	A Randomized Phase I/II Study of Nivolumab in Combination with GM.CD40L Vaccine in Adenocarcinoma of the Lung;<br>Phase I, II	Not yet open for participant recruitment;<br>June 2015;<br>Estimated: December 2020;<br>October 2017	Lung Cancer Adenocarcinoma	Nivolumab;<br>GM.CD40L Vaccine
NCT02523469;<br>John Wrangle / Altor Bioscience Corporation	A Phase IB/II Study of Nivolumab In Combination with ALT-803 In Patients with Pretreated, Advanced, or Metastatic NSCLC;<br>Phase I, II	Recruiting participants;<br>August 2015;<br>Estimated:	NSCLC	Superagonist Interleukin-15 (ALT-803);<br>Nivolumab

		December 2019;<br>June 2017		
NCT02526017;<br>Five Prime Therapeutics, Inc.	A Phase 1a/1b Study of FPA008 in Combination with Nivolumab in Patients with Selected Advanced Cancers;<br>Phase I	Recruiting participants;<br>August 2015;<br>Estimated: August 2019;<br>March 2017	Advanced Solid Tumors	Cabiralizumab, Nivolumab
NCT02543645;<br>Celldex Therapeutics	A Phase I/II, Open Label, Dose-escalation Study of Varlilumab (CDX-1127) in Combination with Atezolizumab (MPDL3280A, Anti-PD-L1) in Patients with Advanced Cancer;<br>Phase I, II	Terminated (Portfolio re-prioritization);<br>September 2015;<br>May 2017;<br>June 2017	Carcinoma Renal Cell, Kidney Diseases, Kidney Neoplasms, Urogenital Neoplasms, Urologic Diseases, Urologic Neoplasms, Neoplasms by Histologic Type, Neoplasms, Clear-cell Metastatic RCC, Melanoma, TNBC, Bladder Cancer, HNC, NSCLC	Varlilumab, Atezolizumab
NCT02554812;<br>Pfizer	A Phase 1b/2 Open-label Study to Evaluate Safety, Clinical Activity, Pharmacokinetics and Pharmacodynamics of Avelumab (msb0010718c) in Combination with Other Cancer Immunotherapies in Patients with Advanced Malignancies;<br>Phase II	Recruiting participants;<br>September 2015;<br>Estimated: February 2020;<br>October 2017	Advanced Cancer	Avelumab, Utomilumab, PF-04518600, PD 0360324
NCT02606305;<br>ImmunoGen, Inc.	A Phase 1b Study to Evaluate the Safety, Tolerability and Pharmacokinetics of Mirvetuximab Soravtansine (IMGN853) in Combination with Bevacizumab, Carboplatin, Pegylated Liposomal Doxorubicin or Pembrolizumab in Adults with Folate Receptor Alpha Positive Advanced Epithelial Ovarian Cancer, Primary Peritoneal Cancer, Fallopian Tube Cancer, or Endometrial Cancer;<br>Phase I	Recruiting participants;<br>November 2015;<br>Estimated: October 2018;<br>June 2017	Epithelial OC, Primary Peritoneal Cancer, FTC, Endometrial Cancer	Mirvetuximab soravtansine, Bevacizumab, Pembrolizumab; Carboplatin, Pegylated Liposomal Doxorubicin
NCT02650713;<br>Hoffmann-La Roche	An Open-Label, Multicenter, Dose Escalation and Expansion Phase Ib Study to Evaluate the Safety, Pharmacokinetics, and Therapeutic Activity of RO6958688 in Combination with Atezolizumab in Patients with Locally Advanced and/or Metastatic CEA-Positive Solid Tumors;<br>Phase I	Recruiting participants;<br>January 2016;<br>Estimated: July 2019;<br>October 2017	Advanced/Metastatic Solid Tumors	RO6958688, Atezolizumab
NCT02659384;<br>European Organisation for Research and	A Phase II Study of the Anti-PD-L1 Antibody Atezolizumab, Bevacizumab and Acetylsalicylic Acid to Investigate Safety and Efficacy of This Combination in Recurrent Platinum-resistant Ovarian, Fallopian Tube or	Recruiting participants;<br>January 2016;<br>Estimated Primary:	Ovarian Neoplasms	Bevacizumab, Atezolizumab; acetylsalicylic acid

Treatment of Cancer - EORTC	Primary Peritoneal Adenocarcinoma;<br>Phase II	January 2021;<br>December 2016		
NCT02665416;<br>Hoffmann-La Roche	An Open-Label, Multicenter, Dose Escalation Phase Ib Study with Expansion Cohorts to Evaluate the Safety, Pharmacokinetics, Pharmacodynamics, and Therapeutic Activity of RO7009789 (CD40 Agonistic Monoclonal Antibody) in Combination with Vanucizumab (Anti-Ang2 and Anti-VEGF Bi-Specific Monoclonal Antibody) in Patients with Metastatic Solid Tumors;<br>Phase I	Recruiting participants;<br>January 2016;<br>Estimated: July 2018;<br>September 2017	Advanced/Metastatic Solid Tumors	RO7009789, Vanucizumab
NCT02715531;<br>Hoffmann-La Roche	An Open-Label, Multicenter Phase Ib Study of The Safety and Tolerability of Atezolizumab (Anti-PD-L1 Antibody) Administered in Combination with Bevacizumab and/or Other Treatments in Patients with Solid Tumors;<br>Phase I	Recruiting participants;<br>March 2016;<br>Estimated: May 2019;<br>August 2017	Solid Tumor	Atezolizumab, Bevacizumab, Vanucizumab;<br>5-FU, Gemcitabine, Leucovorin, Nab-Paclitaxel, Oxaliplatin, Capecitabine, Cisplatin
NCT02722954;<br>OncoMed Pharmaceuticals, Inc. Celgene Corporation	A Phase 1b, Open-Label, Dose Escalation and Expansion Study of Demcizumab Plus Pembrolizumab in Patients with Locally Advanced or Metastatic Solid Tumors;<br>Phase I	Active, not recruiting participants;<br>March 2016;<br>Estimated: January 2018;<br>April 2017	Locally Advanced or Metastatic Solid Tumors	Demcizumab, Pembrolizumab
NCT02737787;<br>Memorial Sloan Kettering Cancer Center	A Phase I Study of Concomitant WT1 Analog Peptide Vaccine with Montanide and GM-CSF in Combination with Nivolumab in Patients with Recurrent Ovarian Cancer Who Are in Second or Greater Remission;<br>Phase I	Recruiting participants;<br>April 2016;<br>Estimated: April 2018;<br>June 2017	OC, FTC, Primary Peritoneal Cancer, Recurrent OC	Nivolumab;<br>WT1 vaccine
NCT02754141;<br>Bristol-Myers Squibb	A Phase 1/2a Study of BMS-986179 Administered in Combination with Nivolumab (BMS-936558) in Subjects with Advanced Solid Tumors;<br>Phase I, II	Recruiting participants;<br>April 2016;<br>Estimated: May 2020;<br>October 2017	Malignant Solid Tumor	BMS-986179, Nivolumab
NCT02760797;<br>Hoffmann-La Roche	An Open-Label, Multicenter, Dose-Escalation Phase Ib Study with Expansion Phase to Investigate the Safety, Pharmacokinetics, Pharmacodynamics, and Therapeutic Activity of Emactuzumab and RO7009789 Administered	Recruiting participants;<br>May 2016;<br>Estimated: July	Solid Tumors	RO7009789, Emactuzumab

	in Combination in Patients with Advanced Solid Tumors;<br>Phase I	2018;<br>October 2017		
NCT02824965;<br>Olivia Newton-John Cancer Research Institute	A Phase I/II Open-label Trial of Intravenous CAVATAK™ in Combination with Pembrolizumab for the Treatment of Patients with Advanced NSCLC;<br>Phase I	Not yet open for participant recruitment;<br>July 2016;<br>Estimated: July 2019;<br>September 2017	NSCLC	Pembrolizumab;<br>CVA21
NCT02826434;<br>Massachusetts General Hospital / OncoPep, Inc.; AstraZeneca	A Phase 1b Study of Safety and Immune Response to PVX-410 Vaccine Alone and in Combination with Durvalumab in Human Leukocyte Antigen (HLA)-A2+ Subjects Following Standard Treatment of Stage II or III Triple Negative BC;<br>Phase I	Recruiting participants;<br>July 2016;<br>Estimated: August 2022;<br>September 2017	BC	Durvalumab;<br>PVX-410 Vaccine, Hiltonol, XBP1-US/XBP1-SP/CD138/CS1 Multipeptide Vaccine PVX-410
NCT02840994;<br>Bavarian Nordic, Inc.	A Phase 1/2 Trial of CV301 in Combination with Anti-PD-1 Therapy Versus Anti-PD-1 Therapy Alone in Subjects with NSCLC;<br>Phase I, II	Recruiting participants;<br>July 2016;<br>Estimated Primary: December 2020;<br>September 2017	NSCLC	Nivolumab;<br>CV301
NCT02843204;<br>Fuda Cancer Hospital, Guangzhou	Combination of Anti-PD-L1 and NK Immunotherapy for Recurrent Solid Tumors;<br>Phase I, II	Recruiting participants;<br>July 2016;<br>Estimated: July 2019;<br>July 2016	Malignant Solid Tumor	Nivolumab;<br>NK cells
NCT02853318;<br>Roswell Park Cancer Institute	A Phase II Evaluation of Pembrolizumab in Combination with IV Bevacizumab and Oral Metronomic Cyclophosphamide in the Treatment of Recurrent Epithelial Ovarian, Fallopian Tube, or Primary Peritoneal Cancer;<br>Phase II	Recruiting participants;<br>August 2016;<br>Estimated: August 2018;<br>August 2017	Fallopian Tube Clear Cell Adenocarcinoma<br>Fallopian Tube Endometrioid Adenocarcinoma<br>Fallopian Tube Mucinous Adenocarcinoma<br>Fallopian Tube Serous Adenocarcinoma<br>Ovarian Clear Cell Adenocarcinoma<br>Ovarian Endometrioid Adenocarcinoma<br>Ovarian Mucinous Adenocarcinoma<br>Ovarian Serous Adenocarcinoma<br>Primary Peritoneal Serous Adenocarcinoma<br>Recurrent FTC Carcinoma<br>Recurrent OC Carcinoma<br>Recurrent Primary Peritoneal Carcinoma	Bevacizumab,<br>Pembrolizumab;<br>Cyclophosphamide<br>Pembrolizumab

			Undifferentiated FTC Carcinoma<br>Undifferentiated OC Carcinoma	
NCT02857920;<br>Fuda Cancer Hospital, Guangzhou	Combination of Bevacizumab and NK Immunotherapy for Recurrent Solid Tumors;<br>Phase I, II	Recruiting participants;<br>August 2016;<br>Estimated: August 2019;<br>August 2016	Malignant Solid Tumor	Bevacizumab;<br>NK cells
NCT02864381;<br>Gilead Sciences	A Phase 2, Open-Label, Randomized Study to Evaluate the Efficacy and Safety of GS-5745 Combined with Nivolumab Versus Nivolumab Alone in Subjects with Unresectable or Recurrent Gastric or Gastroesophageal Junction Adenocarcinoma;<br>Phase II	Active, not recruiting;<br>August 2016;<br>Estimated: August 2019;<br>July 2017	Gastric Adenocarcinoma<br>Gastroesophageal Junction Adenocarcinoma	Andecaliximab, Nivolumab
NCT02873962;<br>Dana-Farber Cancer Institute	A Phase II Study with A Safety Lead-In of Nivolumab In Combination with Bevacizumab for The Treatment of Relapsed Epithelial Ovarian, Fallopian Tube or Peritoneal Cancer;<br>Phase II	Recruiting;<br>August 2016;<br>Estimated: February 2024;<br>July 2017	Peritoneal Cancer, OC, FTC	Bevacizumab, Nivolumab
NCT02879695;<br>NCI	A Phase 1 Study of Blinatumomab in Combination with Checkpoint Inhibitor(s) of PD-1 (Nivolumab) or Both PD-1 (Nivolumab) and CTLA-4 (Ipilimumab) in Patients with Poor-Risk, Relapsed or Refractory CD19+ Precursor B-lymphoblastic Leukemia;<br>Phase I	Recruiting participants;<br>August 2016;<br>Estimated: November 2021;<br>October 2017	B-ALL	Blinatumomab, Ipilimumab, Nivolumab
NCT02886897;<br>Sun Yat-sen University	A Phase II Study of Combinations of Dendritic Cells and Cytokine-induced Killer Cell （D-CIK) Immunotherapy and Anti-Programmed Death-1 In Refractory Solid Tumors;<br>Phase I, II	Recruiting participants;<br>September 2016;<br>Estimated: October 2019;<br>September 2016	Hepatocellular Carcinoma, RCC, Bladder Cancer, CRC, NSCLC, BC	anti-PD-1;<br>D-CIK
NCT02900664;<br>Novartis Pharmaceuticals	Phase Ib, Open-label, Multi-center Study to Characterize the Safety, Tolerability and Pharmacodynamics (PD) of PDR001 in Combination with CJM112, EGF816, Ilaris® (Canakinumab) or Mekinist® (Trametinib);<br>Phase I	Recruiting participants;<br>September 2016;<br>Estimated: January 2020;<br>October 2017	CRC, TNBC, NSCLC - Adenocarcinoma	PDR001, Canakinumab, CJM112;<br>Trametinib (TMT212), EGF816

NCT02947386;<br>Roswell Park Cancer Institute	A Phase I/II Open-Label Study of Nimotuzumab in Combination with Nivolumab in Patients with Advanced NSCLC;<br>Phase I, II	Recruiting participants;<br>October 2016;<br>Estimated: August 2020;<br>July 2017	EGFR Gene Mutation Recurrent, NSCLC	Nimotuzumab, Nivolumab
NCT02950766;<br>Dana-Farber Cancer Institute/ Bristol-Myers Squibb;<br>Oncovir, Inc.	A Phase I Study Combining NeoVax, a Personalized NeoAntigen Cancer Vaccine, with Ipilimumab to Treat High-risk RCC;<br>Phase I	Not yet open for participant recruitment;<br>November 2016;<br>Estimated: September 2022;<br>November 2016	Kidney Cancer	Ipilimumab;<br>NeoVax
NCT02955290;<br>Roswell Park Cancer Institute	A Phase I/II Study of the EGF Vaccine CIMAvax in Combination with the Anti-PD-1 Nivolumab in Patients with Previously Treated Advanced NSCLC;<br>Phase I, II	Recruiting participants;<br>November 2016;<br>Estimated: June 2021;<br>July 2017	ALK Gene Mutation, EGFR Gene Mutation, Recurrent NSCLC	Nivolumab;<br>Recombinant Human EGF-rP64K/Montanide ISA 51 Vaccine
NCT02983045;<br>Nektar Therapeutics	A Phase 1/2, Open-label, Multicenter, Dose Escalation and Dose Expansion Study of NKTR-214 and Nivolumab in Patients with Select Locally Advanced or Metastatic Solid Tumor Malignancies;<br>Phase I, II	Recruiting participants;<br>December 2016;<br>Estimated: October 2018;<br>September 2017	Carcinoma, NSCLC, Renal Cell Melanoma	Nivolumab;<br>NKTR-214
NCT03014804;<br>Jonsson Comprehensive Cancer Center	A Phase II Clinical Trial Evaluating Combination Therapy Using DCVax-L (Autologous Dendritic Cells Pulsed with Tumor Lysate Antigen) and Nivolumab (an Anti-PD-1 Antibody) for Subjects with Recurrent Glioblastoma Multiforme;<br>Phase II	Not yet open for participant recruitment;<br>January 2017;<br>Estimated: October 2020;<br>October 2017	Giant Cell Glioblastoma, Gliosarcoma, Oligodendroglioma, Recurrent Glioblastoma, Small Cell Glioblastoma	Nivolumab;<br>Autologous Dendritic Cells Pulsed with Tumor Lysate Antigen Vaccine
NCT03026166;<br>AbbVie Collaborator: Bristol-Myers Squibb	A Phase 1/2 Study on the Safety of Rovalpituzumab Tesirine Administered in Combination with Nivolumab or Nivolumab and Ipilimumab for Adults with Extensive-Stage SCLC;<br>Phase I	Recruiting participants;<br>January 2017;<br>Estimated: April 2020;<br>October 2017	SCLC	Rovalpituzumab tesirine, Nivolumab, Ipilimumab

NCT03030287; OncoMed Pharmaceuticals, Inc.	A Phase 1b Study of OMP-305B83 Plus Weekly Paclitaxel in Subjects with Platinum Resistant Ovarian, Primary Peritoneal or Fallopian Tube Cancer; Phase I	Recruiting participants; January 2017; Estimated: December 2019; May 2017	OC, Cancer Peritoneal Cancer, FTC	Navicixizumab; Paclitaxel
NCT03038100; Hoffmann-La Roche	A Phase III, Multicenter, Randomized, Study of Atezolizumab Versus Placebo Administered in Combination with Paclitaxel, Carboplatin, and Bevacizumab to Patients with Newly-Diagnosed Stage III or Stage IV Ovarian, Fallopian Tube, or Primary Peritoneal Cancer; Phase III	Recruiting participants; January 2017; Estimated: July 2025; August 2017	OC, FTC, Peritoneal Neoplasms	Atezolizumab, Bevacizumab; Paclitaxel, Carboplatin
NCT03049618; University of Southern California	A Phase IIa Trial of sEphB4-HSA in Combination with Anti PD-1 Antibody (Pembrolizumab, MK3475) in Patients with Non-Small Cell Lung and Head/Neck Cancer; Phase II	Recruiting participants; February 2017; Estimated: March 2020; March 2017	ALK Gene Mutation, BRAF Gene Mutation, EGFR Gene Mutation, HNSCC, Metastatic HNC, Recurrent HNC, Recurrent NSCLC, ROS1 Gene Mutation, NSCLC	Pembrolizumab; Recombinant EphB4-HSA Fusion Protein
NCT03111992; Novartis Pharmaceuticals	Phase I/Ib, Multi-center, Open-label, Study of Single Agent CJM112, and PDR001 in Combination with LCL161 or CJM112 in Patients with Relapsed and/or Refractory Multiple Myeloma; Phase I	Recruiting participants; April 2017; Estimated: June 2020; August 2017	MM	PDR001, CJM112, LCL161
NCT03118349; MabVax Therapeutics, Inc.	Phase I, Open-Label, Multi-Center, Dose Escalation with Expansion Trial of 177Lu Human Monoclonal Antibody 5B1 (MVT-1075) in Combination with a Blocking Dose of MVT-5873 as Radioimmunotherapy in Relapse/Refractory Subjects with Pancreatic Cancer or Other CA19-9 Positive Malignancies; Phase I	Recruiting participants; April 2017; Estimated: December 2018; June 2017	Pancreatic Carcinoma, Tumors That Express CA 19-9	5B1, 177Lu 5B1
NCT03123055; BioClin Therapeutics, Inc.	A Multi-Center, Single-Arm, Open-Label Phase 1b Study of a Novel FGFR3 Inhibitor (B-701) Combined with Pembrolizumab in Subjects with Locally Advanced or Metastatic Urothelial Carcinoma Who Have Progressed Following Platinum-based Chemotherapy; Phase I	Recruiting participants; April 2017; Estimated: March 2019; September 2017	NSCLC, Urinary Bladder Neoplasms, Neoplasm Metastasis	B-701, Pembrolizumab

NCT03136406; NantCell, Inc.	NANT Pancreatic Cancer Vaccine: Combination Immunotherapy in Subjects with Pancreatic Cancer Who Have Progressed on or After Standard-of-care Therapy; Phase I, II	Not yet open for participant recruitment; May 2017; Estimated: December 2018; October 2017	Pancreatic Cancer	Bevacizumab, Avelumab; Cyclophosphamide, Oxaliplatin, Capecitabine, 5-Fluorouracil, Leucovorin, nab-paclitaxe, ALT-803, aNK cells, ETBX-011, GI-4000
NCT03138889; Nektar Therapeutics	A Phase 1b, Open-label, Multicenter Study to Investigate the Safety and Preliminary Efficacy of NKTR 214 and Anti-PD-L1 (Atezolizumab) in Patients with Locally Advanced or Metastatic Urothelial Bladder Cancer or Metastatic NSCLC; Phase I	Recruiting participants; May 2017; Estimated: May 2020; August 2017	NSCLC, Urinary Bladder Neoplasms, Neoplasm Metastasis	Atezolizumab; NKTR-214 (CD122-Biased Cytokine)
NCT03153410; Sidney Kimmel Comprehensive Cancer Center	A Pilot Study of a GVAX Pancreas Vaccine (with Cyclophosphamide) in Combination with a PD-1 Blockade Antibody (Pembrolizumab) and a Macrophage Targeting Agent (CSF1R Inhibitor) for the Treatment of Patients with Borderline Resectable Adenocarcinoma of the Pancreas; Phase I	Not yet recruiting; May 2017; Estimated: September 2019; May 2017	Pancreatic Cancer	Pembrolizumab; Cyclophosphamide, GVAX
NCT03158272; Bristol-Myers Squibb	A Phase 1 Study of Cabiralizumab (BMS-986227, FPA008) Administered Alone or in Combination with Nivolumab (BMS-936558) in Advanced Malignancies; Phase I	Recruiting participants; May 2017; Estimated: November 2020; October 2017	Advanced Malignancies	Cabiralizumab, Nivolumab
NCT03160079; Matthew Wieduwilt, M.D., Ph.D.	A Phase I/II Study of Blinatumomab in Combination with Pembrolizumab (MK-3475) for Adults with Relapsed or Refractory B-lineage ALL with High Bone Marrow Lymphoblast Percentage; Phase I, II	Recruiting participants; May 2017; Estimated: August 2022; September 2017	B-ALL	Blinatumomab, Pembrolizumab
NCT03169777; NantCell, Inc.	NANT CRC (CRC) Vaccine: Combination Immunotherapy in Subjects with Recurrent or Metastatic CRC; Phase I, II	Not yet open for participant recruitment; May 2017; Estimated: March 2019; October 2017	CRC	Avelumab, Bevacizumab, Cetuximab, Nivolumab; Capecitabine, Cyclophosphamide, 5-Fluorouracil (5-FU), Fulvestrant, Leucovorin, Nab Paclitaxel, Lovaza, Oxaliplatin, Radiation:

				Stereotactic Body Radiation Therapy, ALT-803, ETBX-011, ETBX-021, ETBX-051, ETBX-061, GI-4000, GI-6207, GI-6301, haNK cells
NCT03181308; Tracon Pharmaceuticals Inc.	A Phase 1b Dose-Escalation Study of Carotuximab (TRC105) in Combination with Nivolumab in Patients with Metastatic NSCLC; Phase I	Not yet open for participant recruitment; June 2017; Estimated: December 2018; October 2017	NSCLC	Carotuximab, Nivolumab
NCT03190811; Capital Medical University	A Prospective Study of Anti-PD-1 Alone or Combined with Autologous DC-CIK Cell Therapy in Advanced Solid Tumors; Phase I, II	Recruiting participants; June 2017; Estimated: June 2020; June 2017	Neoplasms	Anti-PD-1; DC-CIK
NCT03199040; Washington University School of Medicine	A Randomized Phase 1 Trial of Neoantigen DNA Vaccine Alone vs. Neoantigen DNA Vaccine Plus Durvalumab in BC Patients Following Standard of Care Therapy; Phase I	Not yet open for participant recruitment; June 2017; Estimated: November 2020; October 2017	TNBC	Durvalumab; Neoantigen DNA vaccine
NCT03214250; Parker Institute for Cancer Immunotherapy	Open-label, Multicenter, Phase 1b/2 Clinical Study to Evaluate the Safety and Efficacy of CD40 Agonistic Monoclonal Antibody (APX005M) Administered Together with Gemcitabine and Nab-Paclitaxel with or Without PD-1 Blocking Antibody (Nivolumab) in Patients with Previously Untreated Metastatic Pancreatic Adenocarcinoma; Phase I, II	Recruiting participants; July 2017; Estimated: September 2022; October 2017	Metastatic Pancreatic Adenocarcinoma	APX005M, Nivolumab; Nab-Paclitaxel, Gemcitabine

1) *Abbreviations*: BC, breast cancer; ALL, acute lymphoblastic leukemia; CRC, colorectal cancer; CI, confidence interval; FTC, fallopian tube cancer; HNC, head and neck carcinoma; MM, multiple myeloma; NCI, National Cancer Institute; NSCLC, non-small cell lung cancer; OC, ovarian carcinoma; TNBC, Triple Negative Breast Cancer.
